# Health benefits of fermented foods: a strategic roadmap from the PIMENTO initiative

**DOI:** 10.3389/fnut.2026.1904521

**Published:** 2026-09-18

**Authors:** Mustafa Guzel, Arghya Mukherjee, Ricardo Assunção, Juana Frias, Christèle Humblot, Marta Laranjo, Isabelle Savary-Auzeloux, Birsen Yilmaz, Christophe Chassard, Smilja Praćer, Guy Vergères, PIMENTO Health Benefits and Risks Working Group, Signe Adamberg, Elizabeth Adesemoye, Fojan Agahi, Zeynep Agirbasli, Ecem Akan, Asli Akpinar, Panagiota Alvanoudi, Emilia Alves, Ljupco Angelovski, Elena Arranz, Sultan Arslan-Tontul, Ricardo Assunção, Erfan Bagherzadehsurbagh, Nurcan Bağlam, Cornelia Bär, Simona Lucia Bavaro, Miona Belović, Muzeyyen Berkel Kasikci, Thomas Bintsis, Grégory Bouchaud, Tugce Bulmus-Tuccar, Kathryn J. Burton-Pimentel, Özge Cemali, Marie-Christine Champomier-Vergès, Emilie Chanséaume-Bussiere, Christophe Chassard, Zuzana Ciesarová, Paul Cotter, Hülya Cunedioglu, Biljana Cvetković, Bojana Danilovic, Muriel Derrien, Ioannis Diamantoglou, Emine Dincer, Julieta Domínguez-Soberanes, Gamze Düven, Mounaim Halim El Jalil, Erenay Erem, Tuba Esatbeyoglu, Anthony Fardet, Dominic N. Farsi, Pavli Foteini, Juana Frias, Mario Caruana Grech Perry, Amparo Gamero, Mónica Gandía, Enriqueta Garcia-Gutierrez, Amaury Gérard, Aiswarya Girija, Carmen M. Gonzalez-Domenech, Ziba Güley, Zehra Gulsunoglu-Konuskan, Burcu Gündüz Ergün, Carolina Gutiérrez-Repiso, Mustafa Guzel, Hayriye S. Harsa, Daniel Heine, Elaine Hillesheim, Melanie Huch, Christèle Humblot, Bahtir Hyseni, Aline Issa, Sumeye Kabakci, Minna Kahala, Hatice Kalkan Yıldırım, Aggeliki Kalogeropoulou, Mustafa F. Karagöz, Barçın Karakaş-Budak, Sibel Karakaya, Harun Kesenkas, Erhan Keyvan, Meral Kilic-Akyilmaz, Gabriela Krausova, Ibrahim E. Künili, Mary-Liis Kütt, Marta Laranjo, Sandrine Louis, Jeadran Malagon-Rojas, Fani Th. Mantzouridou, Julie Mardon, Lidia Markiewicz, Cristina Martínez-Villaluenga, Antonia Matalas, Baltasar Mayo, Ryma Merabti, Victoria Meslier, Sandra Mojsova, Isabel Moreno-Indias, Arghya Mukherjee, Eleni Naziri, Anastasios Nikolaou, Daniela Nikolovska Nedelkoska, Anastasia Ntantou, Sine Özmen Toğay, Sebnem Ozturkoglu-Budak, Vasfiye H. Özyurt, Zada Pajalic, Recep Palamutoglu, Demosthenes B. Panagiotakos, Konstantinos Papadimitriou, Alejandra Parada-López, Diana Paveljšek, Elena Peñas, Vincenzo Pennone, Eugenia Pertziger, Smilja Praćer, Reimo Priidik, Milica Prvulovic, Endra Luzha-Pula, Catarina Rosado, Patricia Ruiz-Limón, Pasquale Russo, Helen Saar, José Ángel Salas-Millán, Seppo Salminen, Elisa Salvetti, Dushica Santa, Taner Sar, Inga Sarand, Isabelle Savary-Auzeloux, Else Schneider, Beatriz N. Silva, Valentina Simeunović, Laurencia Songré-Ouattara, Małgorzata Starowicz, Dominic Stoll, Michail Syrpas, Hania Szajewska, Julien Tap, Ayşe Nur Tonay, Thomy Tonia, Aleksandra Torbica, Biljana Trajkovska, Guy Vergères, Bojana Vidovic, Andjela Vukojevic, Oktay Yerlikaya, Aycan Yigit Cinar, Birsen Yilmaz, Arkadiusz Józef Zakrzewski, Zsolt Zalán

**Affiliations:** 1 Department of Food Engineering, Hitit University, Corum, Türkiye; 2 APC Microbiome Ireland, Cork, Ireland; 3 Department of Food Biosciences, Teagasc Food Research Centre, Fermoy, Co., Cork, Ireland; 4 Food and Nutrition Department, National Institute of Health Dr. Ricardo Jorge, Lisbon, Portugal; 5 Institute of Food Science, Technology and Nutrition, Spanish National Research Council (ICTAN-CSIC), Madrid, Spain; 6 QualiSud, Université de Montpellier, Avignon Université, CIRAD, Institut Agro, IRD, Université de la Réunion, Montpellier, France; 7 French National Research Institute for Sustainable Development (IRD), Montpellier, France; 8 MED-Mediterranean Institute for Agriculture, Environment and Development & CHANGE-Global Change and Sustainability Institute, Departamento de Medicina Veterinária, Escola de Ciências e Tecnologia, Universidade de Évora, Évora, Portugal; 9 INRAE, UMR 1019, UNH, CRNH, Auvergne, Clermont-Ferrand, France; 10 Advanced Research Unit on Metabolism, Development and Ageing (ARUMDA), Tata Institute of Fundamental Research, Hyderabad, India; 11 Tata Institute of Fundamental Research Hyderabad, Hyderabad, India; 12 Unité Mixte de recherche sur le Fromage, INRAE, UCA, VetAgro Sup, Aurillac, France; 13 Department of Neurobiology, Institute for Biological Research “Sinisa Stankovic”, National Institute of the Republic of Serbia, University of Belgrade, Belgrade, Serbia; 14 Food Microbial Systems Research Division, Agroscope, Bern, Switzerland

**Keywords:** bioavailability, fermented foods, food characterisation, gut microbiome, health benefits, lactobacilli, strategic roadmap, yogurt

## Abstract

Fermented foods are receiving increasing attention as potentially beneficial components of healthy diets because they provide live microorganisms, fermentation-derived metabolites, and food matrices modified by microbial activity. However, the evidence supporting their health effects remains heterogeneous, making it difficult to translate current knowledge into clear regulatory or public health guidance. Here we present a Strategic Research and Policy Roadmap developed within Working Group 3 of the COST Action “Promoting Innovation of ferMENTed fOods” (PIMENTO) CA20128 to identify priorities for fermented foods research and policy directions in the near future. Using an approach informed by the European Food Safety Authority framework for health claim substantiation, the PIMENTO WG3 initiative synthesised evidence from a dedicated series of 17 published reviews, comprising systematic reviews, meta-analyses, systematic narrative reviews, narrative reviews, and a scoping review, preceded by a position paper setting out the WG3 strategy, covering human studies, mechanistic evidence, food characterisation, bioavailability, and safety. Across these reviews, fermented foods were associated with generally modest, and sometimes neutral, effects on diverse health outcomes. The collective evidence also highlighted recurring strengths of the field, including biological plausibility, broad health relevance, and promising functional targets for selected fermented food categories, alongside persistent challenges related to food characterisation, study heterogeneity, dose–response assessment, mechanistic validation, and long-term human evidence. Safety findings were broadly reassuring in the populations studied, although reporting was inconsistent. We translate these findings into a strategic roadmap for research and policy, reflecting the expert judgement of the author panel rather than a formal consensus procedure, organised across short-, medium-, and long-term horizons, to harmonise fermented food definitions and exposure assessment, standardise product dossiers and analytical methods, strengthen trial design, incorporate mechanistic validation, and align evidence generation with regulatory and public health needs while remaining relevant to diverse stakeholders. Overall, this work provides a structured foundation for the next phase of fermented food research by identifying evidence gaps, methodological priorities, and policy needs required to advance the field toward greater scientific and regulatory maturity. To our knowledge, this represents the first effort of its kind to consolidate evidence and knowledge gaps across the full breadth of fermented foods research into a single, actionable strategic framework.

## 1 Introduction

The previous decade has witnessed a rise in interest in fermented foods (FF), defined as “foods made through desired microbial growth and enzymatic conversions of food components” (1). Beyond its traditional role in producing safe, shelf-stable foods with improved organoleptic properties (2), fermentation is increasingly recognised for its potential health benefits, drawing growing interest from both researchers and consumers (3). This is reflected in the scale of the sector: approximately one third of foods consumed globally are fermented (4), and the global fermented food and beverage market is projected to reach US$1.25 trillion by 2034 (5).

Fermented foods are part of healthy diets (6) with live dietary microbes (7, 8) and their metabolites (9) contributing to the potential health benefits of FF. These health benefits include the pre-digestion of food (10), the synthesis (11) and improved bioavailability (12) of micronutrients, removal of allergens (13) and anti-nutrients (14), regulation of metabolic health (15) and the regulation of physiological processes across all organ systems including the immune (16) and cardiovascular (17) systems, and the gut-brain axis (18). Interestingly, microbes and related metabolites present in FF may influence different health axes directly, as mentioned above, and may also impact human health indirectly by modulating the intestinal microbiota (19). Indeed, the gut microbiota has emerged as a key intermediary in diet-host interactions (20). These developments have further boosted research on the various elements of microbe-associated foods, including FF, prebiotics, probiotics, and postbiotics, among others (21–25). These terms are related but not interchangeable: probiotics are live microorganisms that, when administered in adequate amounts, confer a health benefit on the host; postbiotics are preparations of inanimate microorganisms and/or their components that confer a health benefit on the host (1); and live culture versus pasteurised fermented foods differ specifically in whether viable microorganisms remain present at the point of consumption.

Despite this wealth of scientific information, evidence for the potential health benefits of FF remains difficult to interpret for several reasons. For example, the specific nutritional and health properties of fermented foods have been recognised in the dietary guidelines of only a few countries even though microbe-associated foods (‘microbial foods’) such as FF may help deliver better, tailored dietary advice by taking inter-individual variability (i.e., personalised nutrition) into account (22, 26–29). Additionally, the overall scientific evidence linking the intake of microbial foods, particularly FF as a whole, to human health remains inconclusive at present, with only two health claims having been recognised for FF by regulators, both for yogurt (30, 31); the EU claim is an EFSA-authorised health claim under Regulation (EC) No 1924/2006, while the FDA claim is a qualified health claim with a lower evidentiary tier. Importantly, although FF are generally considered safe for human consumption (32), hazards associated with their microbial or chemical components (e.g., pathogens, toxins, biogenic amines) remain relevant, especially with the development of novel plant-based FF (33), and remain under-researched. Finally, a large gap remains between what is recommended or advertised in the popular press and social media regarding the potential health effects of some FF and their scientific evaluation by expert panels and regulatory authorities.

To advance the integration of FF into dietary guidelines along with tailored recommendations, a strategic research and policy roadmap is needed to produce substantial and reliable long-term evidence. This begins with a systematic evaluation of current scientific evidence and identification of knowledge gaps linking FF intake to human health. This assessment was conducted as part of the COST Action “Promoting Innovation of ferMENTed fOods” (PIMENTO) CA20128 Working Group 3 (34) with a consolidated strategic roadmap presented here. The strategic roadmap aims to synthesise the key collective outcomes from all WG3 activities focusing on (i) adjudicating of the current levels of scientific evidence supporting the health benefits of fermented foods consumption, (ii) identifying key knowledge gaps to be addressed by future research, and importantly, (iii) informing and strategizing potential priority areas for future research expenditure in this field. To this end, the Roadmap discusses key insights from PIMENTO WG3 activities on relevant clinical studies, the quality of these studies, mechanisms of action, bioavailability of bioactive compounds, characteristics of the FF, and their bioactive compounds. Additionally, key insights regarding safety issues or risks associated with fermented foods investigated in WG3 are briefly presented to inform future risk–benefit analyses. An evaluation of the total evidence gathered is also provided and briefly discussed. Finally, the strategic roadmap discusses potential policy recommendations (i) to regulatory and public health bodies for nutritional policies based on the identified evidence as well as (ii) to the scientific community for future research based on the identified gaps, as part of its short- to long-term strategic research vision. To our knowledge, this roadmap constitutes the first comprehensive, cross-domain synthesis of fermented foods research and its associated knowledge gaps, translating evidence spanning human health outcomes, mechanisms, food characterisation, bioavailability, and safety into a unified set of research and policy priorities.

## 2 PIMENTO working group 3: overview and insights

### 2.1 Scope and objectives

The long-term goal of PIMENTO (34) is to position Europe at the forefront of innovation on microbial foods, promoting health, regional diversity of FF, local production at different scales, and contributing to economic development and food sovereignty. This objective is pursued through coordinated research activities including mapping FF production and consumption across COST countries, evaluating health benefits and risks, identifying bottlenecks limiting fermentation-based innovation, and disseminating research outputs along with capacity-building actions linking science to stakeholders, citizens and regulatory bodies.

PIMENTO is organised in five working groups (WG) with WG3 “Health benefits and risks of fermented foods” specifically aimed at evaluating the state-of-the-art in terms of scientific evidence and knowledge gaps in the health benefits and risks of FF consumption. To achieve this goal WG3 is articulated around three major milestones:

- *Milestone 1.* Publication of a position paper on the strategy taken by PIMENTO to provide scientific evidence on health benefits and risks of FF, including open-source publication of the protocols. This article has been published in 2024 (35).
- *Milestone 2*. Publication of a range of 17 review-based publications (hereafter, ‘PIMENTO reviews’), based on the EFSA guidance for stakeholders on health claim application (36, 37), assessing the scientific evidence for health benefits and risks of FF, each of the PIMENTO reviews addressing a specific nutritional or health outcome, including safety (Table 1).
- *Milestone 3.* Publication of strategic roadmap that (i) in the short-term, identifies needs for standardisation and foundation for forwarding fermented foods research, (ii) in the medium-term, clarifies the path for strengthening the evidence for specific health properties of FF, and (iii) in the long-term, leads to the integration of FF as a specific food category and an important and safe part of healthy diets into nutritional public health policies (Figure 1).

**Table 1:** Overview of the PIMENTO reviews.

Project code	PIMENTO WG3 project details
Position paper	Health Benefits and Risks of Fermented Foods -The PIMENTO Initiative Health Benefits and Risks of Fermented Foods -The PIMENTO Initiative (35)
E1	Gastrointestinal health
	§ Does consumption of fermented foods have a beneficial effect on gastrointestinal discomfort and normal defecation in a healthy adult population?
	※ Impact of fermented foods consumption on gastrointestinal wellbeing in healthy adults: a systematic review and meta-analysis (57).
	‡ Fermented food consumption may beneficially impact the frequency of bowel movements, stool consistency, incidence of hard stools, intestinal transit time, abdominal symptoms, bloating, borborygmi, flatulence and degree of constipation.
	◊ Certainty of evidence was highly variable and mostly low.
E2	Allergies
	§ What is the effect of the consumption of fermented foods on the development of food allergic symptoms in the food allergic population and a population at high risk for food allergy?
	※ The role of fermented foods in managing food allergies in children and adults: a systematic review (43).
	‡ Fermented foods hold promise in reducing food allergenicity and promoting tolerance.
	◊ Rigorous, well-designed human clinical trials, complemented by mechanistic studies *in vitro* and *in vivo*, are needed to clarify the role of fermented foods as dietary or even clinical tools to combat food allergies.
E3	Immunity
	§ Can consumption of fermented foods aid in prevention of bacterial vaginosis and/or vulvovaginal candidiasis?
	※ Efficacy of fermented foods for the prevention and treatment of bacterial vaginosis and vulvovaginal candidiasis (49).
	‡ Fermented foods have potential as carriers for beneficial microorganisms that are relevant in supporting vaginal health.
	◊ Following EFSA’s guidance on the substantiation of health claims, the current evidence is “neither convincing nor sufficient” to establish a causal relationship. Well-designed studies with thorough product characterisation are needed to substantiate effects and support potential health claims.
E4	Metabolic health
	§ Does fermented foods consumption help to reduce postprandial glucose response or maintain fasting blood glucose concentrations or insulin sensitivity in healthy or prediabetic adults?
	※ Fermented bread consumption: Effects on glycaemia in healthy populations (45).
	‡ Some evidence is available to suggest a beneficial effect of sourdough compared to yeast-fermented bread on glycaemic and insulinaemic homeostasis in healthy individuals.
	◊ Taking into account the risk of bias of the studies, the consistency of the reported effects, and the biological plausibility based on food characteristics and the potential mechanisms of action, the beneficial evidence available for a beneficial effect of sourdough compared to yeast-fermented bread on glycaemic and insulinaemic homoestasis in healthy individuals is neither convincing nor sufficient.
E5	Cardiovascular health
	§ Does consumption of fermented dairy products impact blood lipids in healthy adults?
	※ Fermented dairy product consumption and blood lipid levels in healthy adults: a systematic review (42).
	‡ A few studies with fermented dairy products reported modest reductions in total cholesterol and LDL cholesterol or improved LDL/HDL ratios, particularly with yoghurt and kefir.
	◊ Overall, study quality, result consistency, and mechanistic evidence were deemed “neither convincing nor sufficient” per EFSA criteria. Key limitations included high risk of bias, heterogeneous designs, inadequate product characterisation, and limited mechanistic data. More rigorous, well-controlled human studies with appropriate comparators are needed to clarify whether conventional fermented dairy products have any lipid-lowering effects.
E6	Bone health
	§ What is the effect of consuming fermented foods on the bone health of healthy adults and adults with osteoporosis and osteopenia?
	※ Impact of yogurt consumption on bone health markers in adults with or without osteoporosis: a systematic review and meta-analysis (44).
	‡ Some intervention studies with yoghurt report modest improvements in bone mineral density and bone biomarkers.
	◊ The overall evidence remains inconclusive, hindered by the heterogeneity in study designs and inconsistent yogurt intake. Current evidence does not support a significant role of yogurt consumption in preventing fractures or improving bone mineral density in adults. Well-designed randomised controlled studies are needed to clarify its effects, particularly in adults at risk of or with osteoporosis or osteopenia.
E7	Cognitive health
	§ Does the consumption of foods fermented with *Lactobacillus* sp. and/or *Bifidobacterium* sp. have a beneficial effect on cognitive performance in a healthy adult population, including adults with mild cognitive impairment?
	※ The effects of *Lactobacillus* and/or *Bifidobacterium* in fermented foods on cognitive health: a systematic review (47).
	‡ Consumption of foods fermented with Lactobacillus and/or Bifidobacterium species may offer promising cognitive benefits, particularly in episodic memory and executive functions.
	◊ Following EFSA’s guidance on the substantiation of health claims, the current evidence is “neither convincing nor sufficient” to establish a causal relationship. Well-designed studies with appropriate controls, follow-up periods, larger sample sizes, dose–response assessment and thorough product characterisation are needed to substantiate effects and support potential health claims.
S1	Bioactive compounds
	§ What compounds derived from food fermentation are associated with effects on clinical endpoints in human studies?
	※ Bioactive compounds in fermented foods: a systematic narrative review (61).
	‡ A total of 31 bioactive compounds and/or compound groups in fermented foods, including bioactive peptides, polyphenols (epicatechin, genistein), γ-aminobutyric acid, acetic acid, curcumin, and arabinoxylans, have been associated with beneficial effects in human studies.
	◊ not assessed
S2	Production of vitamins
	§ Does the consumption of fermented foods, fortified in vitamin by fermentation, contribute to vitamin coverage of a healthy population or of a vitamin-deficient population?
	※ Vitamins Formed by Microorganisms in Fermented Foods: Effects on Human Vitamin Status – A Systematic Narrative Review (59).
	‡ Folate bioavailability is enhanced in some cases following the consumption of Camembert cheese naturally rich in folate, while vitamin K2 status is effectively improved in several studies on natto and in one study on Jarlsberg cheese.
	◊ Despite encouraging data, there is a lack of well-controlled, large-scale human studies to validate fermented food as a sustainable strategy to improve vitamin status. Future human studies research should investigate strain-specific effects, food matrix interactions, and long-term health outcomes.
S3	Bioavailability and bioaccessibility of nutrients
	§ Does sourdough and regular bread fermentation increase iron bioavailability, absorption, and status in humans?
	※ Effects of sourdough- and regular-bread fermentation on iron bioavailability, absorption and status in humans: a systematic analysis of human studies (55)
	‡ Mechanistic evidence supports that the sourdough-fermentation process in bread fabrication improves iron bioavailability through phytate reduction.
	◊ No human studies address the question whether the sourdough-fermentation process in bread fabrication improves iron bioavailability.
S4	Ethnic foods
	§ What are the health effects of ethnic fermented foods?
	※ Health benefits of ethnic fermented foods (51).
	‡ Ethnic fermented foods are important in improving health, emphasising their role in sustainable food systems, and underscoring the importance of preserving traditional practices.
	◊ not assessed
S5	Healthy diets
	§ What is the impact of fermented foods consumption on mortality risk?
	※ A systematic review of prospective evidence linking non-alcoholic fermented food consumption with lower mortality risk (54).
	※ Fermented foods consumption, all-cause, and cause-specific mortality: a meta-analysis of prospective cohort studies (46).
	‡ Habitual consumption of certain fermented foods may be associated with modest reductions in mortality risk. Specific fermented foods consumption, i.e., milks, cheese, and chocolate, might be protective against all-cause and CVD mortality, with additional evidence of a protective effect of fermented milk on overall cancer mortality.
	◊ The current evidence remains insufficient to support EFSA-approved health claims. Randomised controlled trials are essential to demonstrate causality. While long-term trials with mortality endpoints are not feasible, studies targeting intermediate outcomes linked to mortality offer a practical alternative. These should be complemented by observational studies to capture long-term, real-world associations.
S6	Personalised nutrition
	§ Does the impact of fermented foods on different health outcomes depend on specific characteristics of population groups, and, consequently, can fermented foods be used in tailored nutritional strategies?
	※ A scoping review on the health effects of fermented foods in specific human populations and potential use of fermented foods in precision nutrition: knowledge and gaps (48).
	‡ Fermented foods have a potential role in tailored nutritional strategies.
	◊ not assessed
S7	Food safety
	§ What are the main microbiological and chemical hazards posed by fermented foods and their associated risks?
	※ Are fermented foods safe for human health? A systematic analysis of the literature (41).
	‡ Fermented foods are generally safe for human consumption when produced under appropriate hygienic and technological conditions. Identified hazards were mainly linked to contaminated raw materials, inadequate process control, post-process contamination, environmental factors, or associated processing steps rather than fermentation itself.
	◊ The evidence supports a reassuring safety profile, but certainty is limited by heterogeneous product categories, variable production contexts, and incomplete risk characterisation for some hazards and vulnerable groups.
S8	Novel fermented foods
	§ What is the health effect of novel fermented foods?
	※ A Systematic Review on the Health Effects of Fermented Wheat Germ Extract with Emphasis on Cancer (58).
	‡ Bioactive constituents of fermented wheat germ extract (FWGE), particularly benzoquinones, peptides, and phenolic compounds, have potential as mediators of its anticancer and anti-inflammatory properties. Among its proposed mechanisms, FWGE may suppress cancer cell proliferation by disrupting the glucose-related metabolic pathways. FWGE may possess therapeutic potential, especially in oncology.
	◊ The current evidence base is insufficient to draw definitive conclusions, and well-designed clinical trials are needed to strengthen this evidence. Moreover, future research should also explore the development of novel FWGE formulations with enhanced bioactive profiles, optimised by modulating fermentation conditions, including such as microbial strain, pH, temperature, and duration.
S9	Food byproducts
	§ What is the health effect of fermented whey?
	※ A systematic review of health promoting effects of consumption of whey-based fermented products on adults (56).
	‡ Fermented whey products demonstrate promising health benefits across multiple physiological systems, with reported improvements in muscle mass, glycemic control, lipid profiles, immune function, oxidative stress and inflammation, and gastrointestinal and urinary tract health.
	◊ Further large-scale, long-term clinical trials are needed to confirm efficacy and mechanistic hypotheses should be tested in preclinical models and supported in humans using relevant functional biomarkers.

Research domain in relation to fermented foods (in beige box), research question (§), linked publication (※), conclusions from project (‡), and comments on certainty of evidence (◊). This table was compiled from each of the 17 PIMENTO reviews and represents a global overview of the main conclusions drawn by the authors of each review, rather than a basis for cross-review comparison; entries marked “not assessed” indicate reviews that were not evaluated against the EFSA evidentiary framework and should not be read as indicating low or insufficient certainty. Quantitative detail for each review, such as number of studies, review type, search dates, and risk-of-bias tool, is reported in Supplementary Table S1.

**Figure 1:**
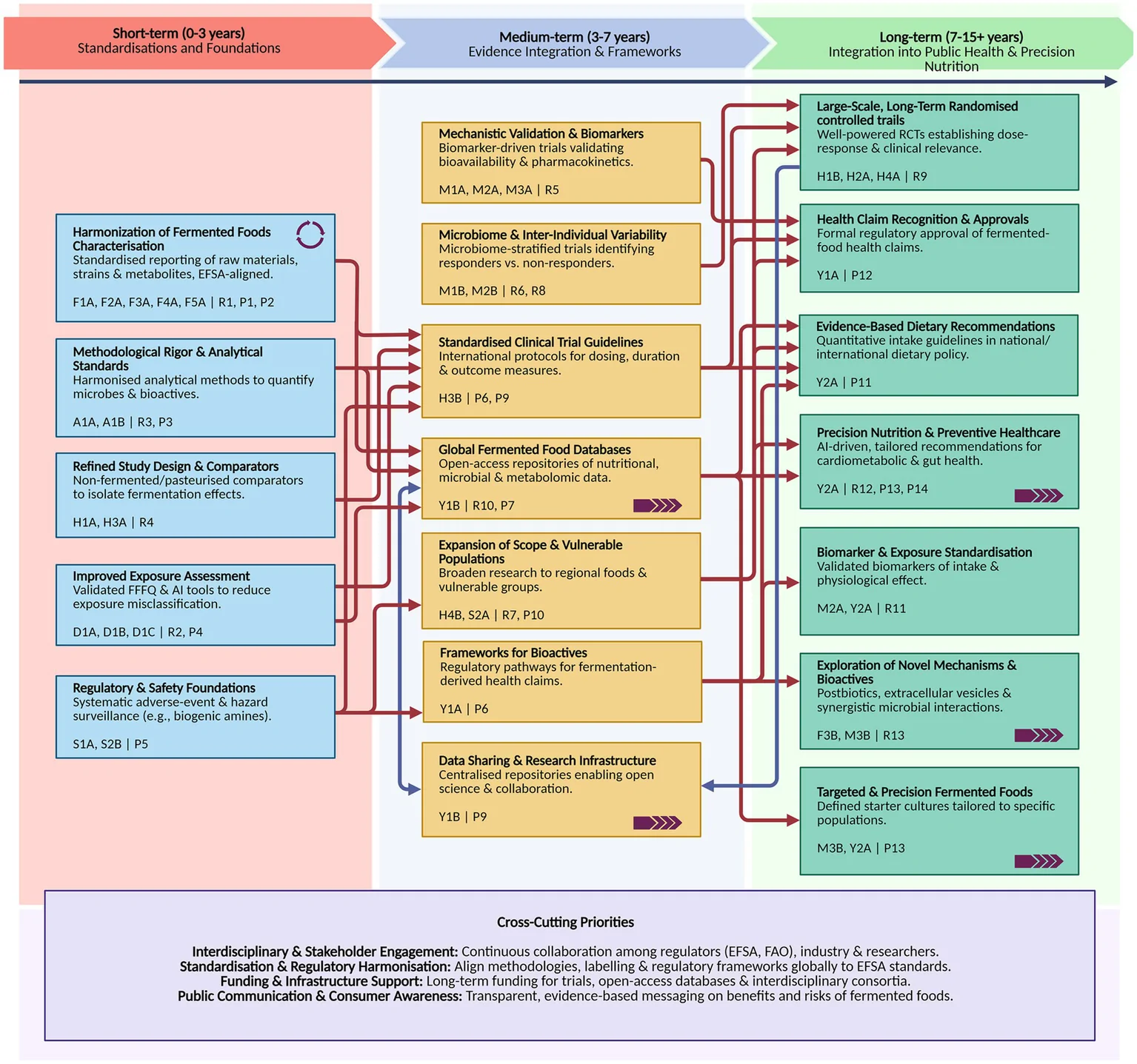
Strategic research and policy roadmap for fermented foods. This roadmap illustrates the strategic transition required to move from fragmented, poorly characterised, association-based evidence in fermented foods research to a more standardised, mechanistically validated, regulatory-grade science and evidence-based public health nutrition. Timeframes for roadmap objectives are mapped on the horizontal axis. Actions are structured across three chronological phases: Short-term (0–3 years) focusing on standardisation and foundational tools, medium-term (3–7 years) targeting mechanistic validation and evidence integration, and long-term (7–15 + years) aiming at large-scale evidence consolidation and precision nutrition. Discrete boxes represent deliverables with connecting arrows (maroon in colour) mapping critical dependencies, demonstrating how foundational actions are prerequisites for building global databases, conducting large-scale clinical trials, and eventually achieving health claim approvals and evidence-based dietary recommendations in the longer term. Certain actions that can act retroactively or are mutually dependent are indicated by blue arrows. The bracketed tags within each action node indicate specific EFSA-guided domain framework items and denote exactly which Research Roadmap (R1-R13) and Policy Roadmap (P1-P14) objectives are fulfilled by that action. Some roadmap goals fulfil both research and policy roadmap objectives and are indicated likewise in the action boxes. The major EFSA-guided Domains as indicated in the roadmap are: Food Characterisation (F), Analytical Methods (A), Dietary Assessment (D), Human Evidence (H), Mechanisms (M), Safety (S), Synthesis & Integration (Y), with the Subdomains being: Raw Materials & Composition (F1), Food Diversity & Cultural Scope (F2), Manufacturing Process (F3), End-Product Profiling (F4), Stability & Delivered Exposure (F5), Analytical Methodologies (A1), Exposure Tracking (D1), Specificity & Magnitude of Effect (H1), Dose–Response (H2), Study Design & Controls (H3), Population & Sustainability (H4), Bioavailability (M1), Causal Linkages (M2), Vitamins (M3), Clinical Tolerability (S1), Vulnerability & Risk (S2), Regulatory Submissions (Y1), Precision Nutrition (Y2). Further details can be found in Supplementary Table S2. Certain roadmap objectives are considered cross-cutting priorities and underpin the entire roadmap across all timeframes; these are shown as a separate domain. These include continuous interdisciplinary stakeholder engagement, transparent public communication, and sustained infrastructure funding, among others. Roadmap actions expected to continue beyond their timeline are indicated by purple progress chevrons at the bottom of the corresponding action box with actions anticipated to be iteratively improved upon are indicated by a purple circular progress icon. To translate these timeframes into concrete, assessable progress, each phase corresponds to measurable milestones with the full underlying detail presented in the newly commissioned Supplementary Figure S1. In the short-term, progress may be measurable through the publication and uptake of a minimum reporting checklist for fermented food characterisation and a standardised fermented-food-trial-conduct guideline, tracked via the proportion of newly registered fermented food trials and reviews that adopt them. In the medium-term, progress is measurable through the proportion of the knowledge gaps identified in this Roadmap that are addressed by biomarker-integrated human trials, and through the growth and utilisation of the proposed open-access Global Fermented Food Databases. In the long-term, progress may be measurable through the number of fermented-food-specific health claims formally evaluated under EFSA/FDA or equivalent frameworks and the inclusion of fermented-food-specific guidance in national/international dietary policy document alongside the maturation of the fermented food database into an integrated, long-term repository linking product characterisation with preclinical and clinical outcome data.

### 2.2 Methodological strategy

The PIMENTO WG3 strategy has been described in detail in the position paper published previously (35). Briefly, 16 thematic areas were selected to investigate the current evidence for FF consumption on health (Table 1). Two publications, S5A and S5B, jointly address the same thematic area (fermented foods and mortality) through complementary review designs (a systematic review of prospective cohort studies and a meta-analysis of prospective cohort studies, respectively), leading to 17 publications mapping onto 16 distinct thematic areas. Among these, each of the seven PIMENTO E-reviews contained the main elements of EFSA health claim dossiers and comprised four sections: (i) systematic review of human studies addressing the review question; (ii) mechanisms of action and bioactive compounds associated with the health outcome including, when appropriate, the mediating role of the intestinal microbiota; (iii) characteristics of the FF used for the intervention/exposure with a focus on the microbial composition and bioactive compounds mediating the health effect; (iv) safety. The 10 PIMENTO S-publications used evidence-synthesis designs, including systematic reviews, systematic narrative reviews, scoping reviews, and structured narrative analyses. Where relevant, these reviews incorporated systematic search and screening procedures for human studies addressing a specific review question, but they were generally less standardised in structure than the PIMENTO E-reviews. Additional sections (ii)-(iv) were included where appropriate.

PIMENTO WG3 aimed to objectively evaluate the relevant scientific evidence for the health benefits and safety of FF. Corresponding guidance published by regulatory authorities for the submission of health claims in Europe (36–38) were taken into account. Health claim dossiers are owned by the submitting entities, mostly private companies, submitting them to the regulatory authorities and are consequently not made public. Academic researchers not pursuing the registration of health claims are thus generally not exposed to this methodology. In this context, the PIMENTO WG3 strategy was to engage researchers of the PIMENTO network in preparing open-source reviews on selected health benefits and risk of FF based on the structure of health claim dossiers. This strategy aimed to improve and harmonise how scientists assess the functionality of FF based on the assessment of the relationship between foods or food constituents and health, which is at the core of health claim dossiers (36, 37).

The methodology for conducting the systematic review of human studies was harmonised by following the 24-step guidance proposed by Muka et al. (39) with study protocols for each PIMENTO review deposited in the Open Science Framework (OSF) (35, 40). In particular, the methodological quality of the human studies investigating FF was considered when evaluating the evidence for their health benefits and risks. This was facilitated by (i) a distinct, well-formulated research question (e.g., the research question should clearly differentiate between the effect of the whole fermented foods and the impact of fermentation of the food matrix on the clinical outcome as this will determine the nature of the control needed for the study), (ii) appropriate consideration for potential confounding factors given the complexity of FF (e.g., the investigated FF should be well characterised and meet minimal standards for strain-level information similar to standards in probiotic development), (iii) availability and use of appropriate food frequency questionnaires, and (iv) identification of evidence on the mechanisms of action and the mediating bioactive components. The systematic review and quality appraisal of studies was followed, in the same article, by non-systematic, non-clinical sections and, finally, by an EFSA-based evaluation of the totality of evidence integrating product characteristics and mechanism of action with the analysis of the human studies. The *summary of the evidence* resulting from this synthesis was expressed, as by the EFSA, with one of the following three phrases: (i) convincing and sufficient; (ii) neither convincing nor sufficient; or (iii) no or very limited evidence. Although this EFSA classification was not applied mandatorily in every PIMENTO review, most reviews used it, or a closely related interpretive approach, to summarise the strength of the totality of evidence. Methodological quality and risk of bias were assessed separately using review-appropriate tools where applicable, rather than by EFSA guidance itself.

#### 2.2.1 Roadmap synthesis methodology

The evidence base synthesised in this Roadmap is closed and pre-defined: the 17 PIMENTO WG3 reviews described above, each conducted under its own registered protocol and pre-specified search or cut-off date deposited on the Open Science Framework (35, 40). No independent literature search was undertaken at the Roadmap level, and all aggregate counts, evidence classifications, and Supplementary Tables S1, S2 entries are drawn directly from these published or accepted PIMENTO WG3 outputs rather than from a separate, Roadmap-specific analysis. Non-PIMENTO literature cited elsewhere in this manuscript is used for background and contextual framing only, not as additional evidence input to the synthesis. The PIMENTO initiative aims at identifying generic research evidence and gaps on the health properties of fermented foods by taking a matrix approach that covered a broad, but not comprehensive, range of specific health outcomes for specific fermented foods (and specific human populations, mostly adults); as such, not all health outcomes for all FF and all specific populations were investigated. Alcoholic fermented beverages in particular were excluded from most PIMENTO reviews and are therefore not represented in this evidence base. As an extension, PIMENTO does not make any claims on the generalisability of its findings but hopes to provide a foundational platform based on extensive PIMENTO groundwork for future fermented foods research. Readers seeking a fuller treatment of fermented-food safety hazards, including biogenic amines, mycotoxins, ethyl carbamate formation, antimicrobial resistance, mobile genetic elements, foodborne pathogens, and risks in immunocompromised populations, are directed to the dedicated WG3 safety review (41).

Synthesis across the 17 reviews was overseen by a Roadmap panel of 10 of the manuscript’s 11 primary authors, who met monthly from December 2024 onwards to define the manuscript’s structure; this structure was subsequently reviewed, and approved by all 141 co-authors of the PIMENTO WG3 initiative. For each section, a primary contributor and an internal reviewer were assigned and jointly agreed a section-specific data-extraction approach, which necessarily varied given the differing designs, i.e., systematic review, meta-analysis, scoping review, or structured narrative analysis, of the underlying reviews (Section 2.2). Each extraction approach was presented to the Roadmap panel for approval before the corresponding section was written, and any disagreement on extraction or interpretation within a section was resolved by referral to the panel. Extracted findings were then mapped to knowledge gaps and to the Research and Policy objectives set out in Section 3, with the full mapping detailed in Supplementary Table S2. This process reflects the expert judgement of the Roadmap panel and the wider PIMENTO WG3 author group; it did not follow a Delphi or other formal structured procedure for group agreement, nor was it registered with the EQUATOR Network, consistent with the Roadmap’s nature as an expert-perspective opinion document rather than a set of proposed standards, recommendations, or guidelines.

### 2.3 Summary of results

#### 2.3.1 Systematic review of human studies

The PIMENTO WG3 initiative culminated in publication of 18 total outputs: one position paper and 17 review-based publications (including 3 meta-analyses and 4 systematic narrative or scoping reviews), which collectively addressed the central question: *is there evidence to support an association between the consumption of FF, either as a group or as specific types, and beneficial nutritional or health outcomes?* Furthermore, if such evidence exists, is it robust enough to justify the submission of a nutritional or health claim for EFSA evaluation? The health outcomes and research domains addressed in the PIMENTO initiative are detailed in Table 1 and include gastrointestinal health, allergy, immunity, metabolic and cardiovascular health, bone health, glycaemia, cognitive health, mortality, micronutrient status, bioactive compounds, nutrient bioavailability, ethnic fermented foods, personalised nutrition, safety, novel fermented foods, and fermented by-products (42–49).

A detailed summary of the main effects and evidence of potential beneficial effects of FF on the outcomes cited above are summarised in Supplementary Table S1. Readers seeking a more granular, review-specific characterisation of individual fermented food categories by food type, microbial composition, fermentation process, and microbial viability are directed to the corresponding individual PIMENTO review (Table 1). From the 17 PIMENTO reviews, a total of 651 human studies were analysed. This total reflects review-level study inclusions rather than a deduplicated count of unique studies, since a study addressing multiple outcomes may be counted in more than one PIMENTO review; the same caveat applies to the proportions of interventional and observational studies below. Among these human studies, the majority were interventional studies while the remaining were observational studies, ranging in size from a few dozen to tens of thousands of participants. The study participants were from different countries worldwide, with an underrepresentation of South America and an overrepresentation of Westernised countries and Asia. In contrast, human studies specifically targeting ethnic fermented foods were mostly from Africa and Asia despite the existing knowledge of FF consumption worldwide (50, 51). The fermented foods most frequently covered by the PIMENTO reviews were overall FF, fermented dairy products, fermented cereals (e.g., bread), fermented legumes (e.g., soy-based products) fermented vegetables, and chocolate. Alcoholic fermented beverages were excluded from nearly all component reviews at the eligibility-criteria stage (e.g., the WG3 Safety review, S7, excluded beverages above 1.25% alcohol by volume), rather than only from this synthesis. Coffee and tea were treated inconsistently across the component reviews rather than uniformly excluded: tea is, with limited exceptions, not itself a fermented product and was therefore out of scope by definition, while coffee undergoes a fermentation step during processing but was addressed only by a subset of reviews (e.g., the WG3 Scoping review, S6, which discussed coffee and tea separately from the core FF categories) rather than treated as a target FF category throughout. The fermented foods categories summarised here thus predominantly refer to non-alcoholic FF. Although a broad range of fermented foods was investigated in these human studies, a few FF and functionalities are overrepresented. A representative example over the last decades is dairy products. Their role in the development of obesity, diabetes, and cardiovascular diseases (CVD) has been widely investigated and debated. The controversies surrounding dairy products, largely due to their fatty acid content and composition, have indeed led to the publication of a substantial body of research on their health effects across different populations. In this context, a central focus of these investigations has been the distinction between fermented and non-fermented dairy products (e.g., cheese, yogurt, fermented milk, kefir) (52, 53).

The conclusions drawn across the PIMENTO reviews were generally consistent: even if increased consumption of fermented foods, either as a whole or as specific types, may be associated with generally modest beneficial effects (sometimes neutral, rarely detrimental) on various health outcomes, current evidence is not sufficient to support an objective claim in relation to a specific outcome or a specific FF. Still, the degree of evidence and reliability of the conclusions vary depending on the FF studied and the outcomes measured. Among the PIMENTO reviews covering a large number of studies, Paveljšek et al. (54) and Matalas et al. (46) reviewed 52 observational studies on the association between total or specific FF intake and mortality risk (all causes, CVD, and cancers). Both PIMENTO reviews showed that even if the effect is modest, fermented dairy, and particularly fermented milk and yogurt are associated with a lower risk of all causes of mortality as well as CVD and cancer mortality, with a high and moderate level of evidence. Cheese is associated with reduced all-cause mortality in some studies but shows inconsistent effect on cardiovascular mortality. Chocolate is also associated with a decreased risk of mortality (all causes and CVD) with a moderate level of evidence. For the other FF studied, even if a slight beneficial effect can be observed for fermented cereals (bread for instance on mortality risks), the levels of evidence range between low and very low. For mortality and cardiometabolic outcomes, meta-analyses indicate small inverse associations between fermented dairy intake and all-cause or cardiovascular mortality, with low to moderate certainty. Of note, effects on lipid profiles and blood pressure were inconsistent across trials (42). For food allergies, Hyseni et al. (43) reported that fermented foods may reduce allergenicity or support tolerance in some contexts, particularly through protein hydrolysis and immunomodulatory pathways, but the available human evidence remains limited and requires better-controlled clinical trials. For metabolic health, Starowicz et al. (45) focused on fermented bread and glycaemic outcomes and concluded that some evidence supports a beneficial effect of sourdough compared with yeast-fermented bread on glycaemic and insulinemic responses, although the overall evidence remains neither convincing nor sufficient. For nutrient bioavailability, Nikolaou et al. (55) reported mechanistic support for sourdough fermentation improving iron bioavailability through phytate reduction, but direct human evidence addressing iron absorption and status remains insufficient. Humblot et al. (48) reported that responses to fermented foods may vary according to population characteristics, baseline health status, and gut microbiota composition, supporting the relevance of fermented foods to future personalised nutrition strategies.

Fermented dairy products, particularly due to their mineral and micronutrient content, have also been studied in populations at risk of osteoporosis. Mayo and colleagues examined the impact of yogurt intake on markers of bone health in 12 observational studies (six included in meta-analysis) (44). Although some studies reported modest improvements in bone mineral density and bone biomarkers following yogurt intake, the meta-analysis showed a neutral effect of yogurt intake on the hazard ratio of hip fracture risk. Hence, the certainty of evidence for a beneficial effect of yogurt on hip fracture and bone mineral density remains low. The consumption of natto or cheese enriched in vitamin K during fermentation has also been shown to promote bone formation and may help maintain skeletal integrity, an effect attributed to the role of vitamin K in osteocalcin activation and improved calcium retention.

Other fermented dairy foods have been specifically investigated for their functionality and potential beneficial effects on various health outcomes. This includes whey-based fermented products, which are traditionally considered by-products of dairy production and have a rich nutritional profile suggesting a potential generation of health-promoting bioactive compounds such as peptides, during fermentation (56). Twelve interventional studies were reviewed that tested the impact of these products on multiple outcomes such as immune function, inflammation, metabolic parameters (glycemia, lipids, etc.), and gastrointestinal health. Although positive effects were recorded at metabolic, cardiovascular and gastrointestinal levels, the number of studies investigating the same clinical indication remains insufficient to allow robust inferences about a positive effect of these whey-based fermented products on any of the various health outcomes tested.

Gut health and gastrointestinal wellbeing (including parameters associated with transit, gastrointestinal symptoms, and stool consistency) are commonly studied in relation to diet composition and nutrient content. A body of 25 human studies was systematically analysed by Mukherjee et al. (57). Despite the significant number of studies, the evidence remains inconsistent, mainly because the parameters used to evaluate the health effects were often too crude (e.g., broadly defined abdominal pain, non-specific severity scales for bloating) to allow consistent conclusions. Notably, the majority of the studies included were RCTs (*n* = 19) where consumption of FF was more controlled, but this was not sufficient to highlight significant and consistent effect. Lastly, the specific role of *Lactobacillus*- or *Bifidobacterium*-based fermentation was evaluated in relation to cognitive health (21 studies reviewed, 8 interventional, 13 observational) (47). Most studies reported beneficial effects on cognition, particularly in episodic memory, executive function, attention, language, verbal fluency, and global cognitive performance. However, when evaluated using an EFSA framework, the evidence is insufficient to establish a consistent causal relationship between FF consumption and cognitive function due to methodological heterogeneity, limited study rigour, inadequate or absent control groups, insufficient information on product characteristics and bioavailability, limited dose–response assessment, and differences in the microbial species and strains used across studies.

Human studies demonstrating the efficacy of FF against bacterial vaginosis and vulvovaginal candidiasis focus exclusively on fermented dairy products (primarily yogurt) with probiotic bacteria as active components (49). These FF all show somewhat robust positive effects, but controls are unclear, and the evidence remains limited. Even though the bibliography investigated only the potential role of fermented dairy products, the number of studies (n = 6) is clearly insufficient to provide objective evidence.

Ethnic fermented foods originating from the heritage and culture of various ethnic groups who utilise their ethno-microbiological knowledge of fermentation with local plant- or animal-based ingredients encompass a wide range of FF such as amazake, boza, buttermilk, injera, kimchi, miso, natto, and tempeh derived from dairy products, fish, meat, legumes, cereals, fruits and vegetables and are deeply rooted in traditional practices. The PIMENTO review by Santa *et al*. investigated the effect of ethnic FF consumption on all human groups from infants to elderly people, healthy or sick and almost all studies were from Asia and Africa (51). The 125 studies included observational as well as interventional studies, covering many outcomes including gut health, immune modulation, metabolic benefit, cognitive function, cardiovascular, bone, oral, eye, skin health, and antimicrobial effect. Potential health benefits were reported across the studies reviewed, and the benefits arose not only from raw ingredients or probiotic content, but from the fermentation process itself, which enhances nutrient bioavailability, reduces antinutrients, and generates bioactive compounds such as amino acids, peptides, and vitamins (K2, B12, riboflavin, folate), highlighting the unique role of fermentation in generating health-relevant components that would not emerge without this biochemical transformation. Importantly, the potential risks of ethnic FF consumption were not investigated in detail and should be taken into account in future studies (51).

One PIMENTO review focused on a functional FF, fermented wheat germ extract (FWGE), which showed association with improved survival and quality of life in people treated for different types of cancer (colorectal, melanoma, neck–head, and prostate), although with a maximum of two publications per cancer type investigated. The potential bioactive constituents of FWGE include benzoquinones, peptides, and phenolic compounds, among others, as mediators of its anticancer and anti-inflammatory properties. The findings suggest that FWGE may possess therapeutic potential, especially in oncology, but the strength of evidence remains limited (58).

Despite the large number of publications commenting on the increase of vitamin content in fermented foods due to microbial synthesis, only a few vitamins (K, B1, B2, B3, B5, B6, B9, and B12) were evaluated in 18 publications for their actual contribution to vitamin status in humans (59). Data on specific vitamins in specific FF is, consequently, limited to a small number of studies, such as for vitamin K in natto (eight publications) and cheese (two publications), and vitamin B9 in cheese (two publications), making generalisation difficult. Of note, populations most at risk of vitamin deficiencies (young children, women of childbearing age, elderly populations) have not been specifically investigated in relation to fermented foods consumption. The investigation of vitamin bioavailability from FF is further complicated by multiple factors, including the microbial strains involved, fermentation conditions, the vitamers produced, and the food matrix, which are rarely assessed comprehensively in the existing literature. There is a notable lack of sufficient studies on several important vitamins, such as vitamin B12. This gap in research data on B12 is especially relevant for populations consuming little or no animal-derived products, where the potential contribution of fermentation could be important. Linked to this lack of human studies, failure to consider fermentation-derived contributions to vitamin production in food may lead to misestimation of vitamin intake (60).

#### 2.3.2 Mechanisms of action and bioavailability of bioactive compounds

Unlike conventional foods, FF provide live microorganisms, fermentation-derived metabolites, and matrices structurally altered by microbial enzymatic activity (1, 61). One PIMENTO review by Künili et al. (61) provided the synthesis of fermentation-derived bioactive compounds, cataloguing 31 compounds associated with clinical outcomes in human studies. These features confer multiple layers of bioactivity, with mechanistic evidence now supporting several domains of action (62) and discussed as a summary below as evidenced in the PIMENTO reviews.

##### 2.3.2.1 Mechanisms based on production of bioactive compounds

Fermentation transforms raw food substrates into complex systems enriched with bioactive compounds. During fermentation, microorganisms, particularly lactic acid bacteria (LAB), yeasts, and propionic acid bacteria, metabolise carbohydrates, proteins, and lipids into molecules that can directly influence human physiology (50, 57). In the gut, organic acids produced during fermentation act as signalling molecules that support gut homeostasis; short-chain fatty acids (SCFAs) such as acetate, propionate, and butyrate are generated separately via a distinct, microbiota-mediated fermentation process in the colon (63). These SCFAs regulate intestinal motility, energy metabolism, and immune homeostasis. Butyrate, for instance, strengthens epithelial tight junctions, enhances mucin secretion, and supports differentiation of regulatory T cells, contributing to gut barrier integrity and reduced inflammation, effects supported mainly by preclinical and mechanistic evidence with limited direct human biomarker confirmation (46, 54, 57, 64). Additionally, proteolysis during fermentation can release short peptides from food proteins with reported biological activities. Among the best characterised are angiotensin-converting enzyme (ACE) inhibitory peptides, such as Val-Pro-Pro (VPP) and Ile-Pro-Pro (IPP), which have been identified in some fermented dairy matrices and can, in principle, modulate blood pressure via the same pathway targeted by ACE-inhibitor drugs (42, 65). Other peptide fractions have been reported to exert antioxidant, antimicrobial, or immunomodulatory effects by scavenging free radicals, modulating cytokine release, or interacting with gut receptors (66). Certain FF can also accumulate γ-aminobutyric acid (GABA) through microbial decarboxylation of glutamate. GABA-enriched fermented products (e.g., some kimchi, yogurt, and fermented soy preparations) have been linked to anxiolytic, antihypertensive, and neuroprotective effects primarily in *in vitro* and animal studies (47, 67). However, given GABA exposure in humans is variable and low, where potential benefits are reported, they may reflect indirect pathways (e.g., modulation of the microbiota–gut–brain axis, co-occurring metabolites, or changes in the food matrix) rather than direct absorption of GABA as a neurotransmitter (68).

Microbial fermentation also enhances the vitamin content of foods, particularly B-group vitamins and vitamin K2 (menaquinone) (59, 69). LAB and propionic acid bacteria can synthesise riboflavin (B2), folate (B9), and cobalamin (B12) in fermented foods, although not all microbially produced corrinoid compounds are biologically active in humans. Certain *Lactococcus* and *Bacillus* species produce menaquinones during cheese and soybean fermentation (51). Vitamin K2 contributes to bone mineralisation through osteocalcin carboxylation and prevents vascular calcification (70), while B12 and folate are critical for one-carbon metabolism. These fermentation-derived vitamins may be nutritionally relevant for specific populations, for example individuals with low intake of animal-derived diets, for whom vitamin B12 deficiency is a particular concern.

Fermentation can also generate or concentrate complex polysaccharides and modify phenolic compounds into potentially more bioaccessible forms. Kefiran, an exopolysaccharide from kefir, has been reported to exhibit prebiotic, immunomodulatory, and anti-inflammatory properties in experimental models (57, 71). In fermented plant-based foods, microbial enzymes may release bound phenolics, increasing antioxidant capacity and free radical scavenging activity (72). These molecules collectively may enhance host antioxidant defence and contribute to the anti-inflammatory and cardiometabolic benefits observed with FF consumption. However, *in vitro* antioxidant activity does not necessarily translate into *in vivo* efficacy, and human studies rarely quantify relevant metabolites or demonstrate systemic exposure. These pathways should therefore be framed as plausible mechanisms requiring targeted, biomarker-informed validation in humans.

##### 2.3.2.2 Mechanisms based on nutrient bioavailability

Fermentation substantially improves the bioavailability of nutrients through several ways including matrix transformation, and degradation of anti-nutritional factors (73). Mechanistically, microbial enzymatic activity can increase nutrient accessibility, alter starch and protein structure, and change the physicochemical environment of the food matrix in ways that may affect digestion and absorption. In cereals, for example, sourdough fermentation by LAB and yeasts can modify starch structure and organic acid profiles, potentially attenuating postprandial glycaemic response (74), while in legumes and vegetables, partial breakdown of cell-wall components may increase bioaccessibility of micronutrients and phytochemicals. Similarly, in dairy products, proteolysis can yield smaller peptides and amino acids that may be more readily absorbed, although the extent and relevance of these changes are product- and process-dependent (75, 76). Fermentation can enhance mineral bioavailability by reducing the content of chelating compounds such as phytates and tannins. Microbial phytases hydrolyse phytic acid, releasing bound iron, zinc, and calcium for absorption (77). Organic acids produced during fermentation may further increase mineral solubility and promote passive absorption in the intestine. For example, fermentation of cereals and legumes has been shown to increase non-haem iron bioavailability, and fermented dairy products have been proposed to similarly improve calcium uptake relative to unfermented milk, although this remains a mechanistically plausible rather than consistently demonstrated effect. These effects are mechanistically attributed to acidification and chelation that favour ionic forms of minerals suitable for absorption.

Beyond the vitamin synthesis itself, fermentation may influence vitamin bioavailability, but direct human evidence remains limited (59). Riboflavin and cobalamin produced by LAB remain more stable during gastrointestinal transit, while vitamin K2 in fermented dairy and soy products may show improved bioaccessibility relative to synthetic forms, although direct human comparative-bioavailability evidence remains limited.

Fermentation can additionally modify protein structures, potentially improving amino acid availability and reducing allergenicity (43). For example, proteolysis occurring during sourdough fermentation can reduce the abundance of specific immunogenic gluten peptides (78), reflecting a reduction in allergenic epitope load rather than a reduction in coeliac disease risk. Allergenicity (an IgE-mediated hypersensitivity response) and coeliac disease (a T-cell-mediated autoimmune response to gluten) are mechanistically distinct, and standard sourdough fermentation does not on its own establish gluten safety for individuals with coeliac disease. This would require a validated process shown to reduce gluten in the final product below the applicable regulatory threshold (e.g., <20 ppm for a “gluten-free” claim under EU Regulation (EU) No 828/2014), a boundary condition not evaluated by the cited evidence. Similarly, soy fermentation reduces allergenic epitopes and improves digestibility (77).

##### 2.3.2.3 Mechanisms mediated by the gut microbiota

Fermented foods can directly and indirectly modulate the gut microbiota, contributing to improved intestinal and systemic health (48, 57, 79). For example, many FF contain live microorganisms that can transiently colonise the human gut (1). Consumption of yogurt, kefir, kimchi, or other fermented products have shown to increase microbial diversity and enrich beneficial taxa such as *Faecalibacterium*, *Roseburia*, and *Bifidobacterium* (80) by providing substrates for these beneficial gut species. These microbes produce metabolites that maintain mucosal health, compete with pathogens, and influence host metabolism. Even pasteurised fermented foods, though lacking live microbes, can modulate the microbiome indirectly through postbiotic molecules and substrate effects. For example, microbial components of FF interact with intestinal immune cells via surface molecules such as lipoteichoic acids and peptidoglycans stimulating innate immune receptors and promoting regulatory immune pathways (57, 81), among others. In both human and animal studies, fermented foods consumption has been associated with increased production of anti-inflammatory cytokines and regulatory T-cell differentiation (82).

SCFAs are key mediators of the microbiota–host interaction. By consuming FF rich in fermentable substrates, individuals enhance microbial SCFA production in the colon by gut microbes. Acetate and propionate contribute to lipid and glucose metabolism, while butyrate serves as a major energy source for colonocytes and suppresses inflammation (83) via G-protein-coupled receptor signalling. SCFAs also stimulate serotonin release, influencing intestinal motility, and improve barrier integrity by upregulating tight junction proteins (84). Notably, improvement of intestinal barrier integrity is not peculiar to SCFAs, with FF-derived microbes shown to enhance mucin production and tight junction integrity, improving gut barrier function.

##### 2.3.2.4 Other mechanisms

Fermented foods contain non-viable microbial components collectively termed postbiotics, that exert biological effects (85). These include bacterial cell wall fragments, secreted peptides, and exopolysaccharides that can modulate immune and epithelial function even in the absence of live microbes. Furthermore, extracellular vesicles (EVs) released by beneficial bacteria during fermentation may serve as nanoscale delivery systems for bioactive molecules, influencing cellular signalling beyond the gut (86). Most supporting data derive from *in vitro* or animal models and therefore require cautious interpretation in the context of human health.

##### 2.3.2.5 Nutritional and clinical indications with incomplete mechanistic support

Despite known bioactives, many observed benefits of FF lack fully elucidated mechanisms (1). Some notable examples include:

- *Constipation relief:* Fermented dairy and kefir improve stool frequency and consistency, but relative contributions of SCFAs, serotonin, and peptides remain unclear (57, 87).
- *Cardiovascular effects:* Yogurt and cheese consumption has been associated with lower blood pressure and cholesterol in some studies, though the evidence is inconsistent and systemic detection of the putative bioactive peptides has not been reliably demonstrated (42, 88). Additionally, populations consuming fermented soy products (e.g., miso, natto) often have lower rates of hypertension and stroke. While some effect may be due to vitamin K2 and its role on arterial stiffness, the role of other fermented compounds, such as nattokinase enzyme or unidentified peptides, in clot prevention and vascular health remains unresolved (46, 51, 54, 89, 90).
- *Bone health:* Yogurt consumption improves calcium balance and reduces bone resorption markers, but vitamin K2 and SCFA bioavailability have not been systematically measured (44, 91).
- *Cognitive outcomes:* Potential improvements in anxiety and mood have been reported with probiotic fermented milks. While GABA and SCFAs offer plausible pathways, the complexity of the gut-brain axis means the precise neuroactive mediators from FF are not fully defined (92). Direct central effects of dietary GABA should be interpreted cautiously, since GABA has limited blood–brain barrier permeability and GABA exposure is rarely quantified in human studies. SCFAs, in contrast, can cross the blood–brain barrier and may influence neuroinflammation, intestinal and neural barrier function, neurotransmission, and Brain-Derived Neurotrophic Factor (BDNF) pathways. Importantly, fermented soy and dairy increase BDNF and improve memory in animal and some human studies, but typical GABA intakes are below mechanistic thresholds (18, 47, 57).
- *Anticarcinogenic properties:* Fermented milk and kimchi have shown anti-tumour effects in cell and animal studies. However, in human epidemiology, it is unclear which components drive any risk reduction. Possible candidates include isoflavones converted by fermentation and bioactive polysaccharides, but definitive links are lacking (51, 93, 94).
- *Immune function:* Several FF reduce allergy and eczema symptoms in children. The immunomodulatory effect may involve modulation of gut microbiota and short-chain fatty acid production, but the chain of causation (which organism or metabolite triggers which immune cell) is still under investigation (95, 96).

##### 2.3.2.6 Mechanisms with preclinical evidence but limited human data

Substantial mechanisms proposed for FF are supported predominantly by *in vitro* experiments and animal models, with limited confirmation in humans. For example, cell-culture studies have shown that extracts of fermented cabbage (kimchi) suppress inflammatory gene expression in intestinal cells (51, 97, 98). Similarly, germ-free mice colonised with microbes from human kefir drinkers showed immune modulation (47, 99). However, these findings do not always replicate in human trials. For instance, many antioxidant compounds identified in FF show strong activity *in vitro* but have poor bioavailability *in vivo* (100).

#### 2.3.3 Characteristics of the fermented foods and their bioactive compounds

##### 2.3.3.1 Source and composition of raw materials

Fermented foods span diverse plant and animal matrices and rely on microorganisms associated with these substrates, or on microorganisms inoculated as starter cultures (47, 48, 51). Common bases include dairy products (yogurt and cheese), grains (sourdough), vegetables (pickles, kimchi, and sauerkraut), legumes (miso, tempeh, and natto), and by-products such as whey (47, 56). In addition, other substrates are used in the production of fermented foods and beverages such as vinegar, cocoa and chocolate, tea, coffee, and a wide range of ethnic FF that represent important elements of cultural heritage (47, 51). Starowicz et al. (45) reported that fermented cereals, particularly rye, oat, and wheat were commonly used as base materials and that product descriptions often specified the grain type, percentage composition, and fermentation agents used. LAB predominate, but yeasts, moulds, and *Bacillus* spp. are central to specific foods (47, 59).

##### 2.3.3.2 Manufacturing processes

Process parameters strongly influence the characteristics of FF (47, 51). For example, longer fermentation and ageing, such as extended cheese ripening, can reduce allergenic epitopes (43) and degrade phytate in sourdough (55). Temperature, oxygen, pH, and duration also influence these profiles. For example, yogurt incubated at 37 °C with LAB produces lactic acid, acetaldehyde, and short-chain fatty acids with viable cells, whereas a whey drink fermented with specific LAB at a controlled pH and then chilled may retain higher levels of amino acids, peptides, and exopolysaccharides with lower number of viable cells (56). Some studies intentionally used techniques to boost health-relevant components such as employing LAB to increase γ-polyglutamate in natto (61).

##### 2.3.3.3 Composition of end products

The final composition of FF reflects a dynamic interaction between the raw material, the microbial community, and the specific fermentation conditions applied. Consequently, the nutritional and biochemical profile of the end product may differ substantially from that of the unfermented matrix. The pairing of raw materials and microbes shapes the nutritional and functional profiles. Soybeans fermented with *Bacillus subtilis* (natto) provide menaquinone-7 (vitamin K2), which is absent in raw soybeans (51, 59). Milk fermented with *Propionibacterium* spp. yields vitamin B12 and K2 (MK-9). LAB fermentation of cereals can increase thiamine (vitamin B1) and riboflavin (vitamin B2) levels (59). Overall, while source-microbe combinations can yield distinctive bioactive profiles, explicit documentation of raw materials and fermentative organisms, including a distinction between live cultures or pasteurised post-fermentation (44), is often incomplete (42, 61).

Fermentation-generated constituents include bioactive peptides, organic acids, bacteriocins, exopolysaccharides, short-chain fatty acids, and vitamins (47, 59, 61). Microbial cells can also be part of the active matrix (57). As reported by the S1 synthesis, 31 bioactive compound classes were linked to human outcomes, such as ACE-inhibitory peptides, γ-aminobutyric acid (GABA), polyphenol aglycones (e.g., genistein) and derivatives, vitamins K2, folate, and B12 (61). These compounds are associated with diverse health effects, such as blood pressure and neurocognition (peptides, GABA), metabolic and antioxidant effects (polyphenol metabolites), and vitamin status (61). Several PIMENTO reviews provide additional examples. For example, in soy-based ferments, such as natto, *Bacillus subtilis* drives the accumulation of vitamin K2 (MK-7) and poly-γ-glutamate, with human studies showing improvements in vitamin K status and bone health markers. Certain cheeses fermented with *Propionibacterium freudenreichii* accumulate vitamin K2 (MK-9) and B vitamins, which were linked to changes in vitamin K status (47, 59). Fermented vegetables provide live LAB populations along with elevated levels of B-vitamins and phenolics compared to raw cabbage (51). Fermented whey beverages deliver peptides, organic acids, and antioxidant constituents, with human trials associating them with beneficial effects on muscle, lipid metabolism, immune activity, and oxidative stress (56). Another example is fermented wheat germ extract, contains benzoquinones that have been investigated for their anticancer potential (58).

##### 2.3.3.4 Stability of end products

Across the PIMENTO reviews, fermented products were typically stored under refrigerated conditions and often consumed fresh, but explicit shelf-life data were rarely reported. Where provided, several beverages and dairy products delivered high viable counts (e.g., ~10^8^ CFU/mL); however, most trials did not re-measure viability at the point of consumption, limiting the interpretation of dose over time (47, 56, 57). In addition, post-fermentation processing steps, such as pasteurisation, filtration, drying or storage, can alter microbial viability and metabolite stability, which shifts the end product characteristics from a fermented food with live microbes to a FF with non-viable microbes. The need to document viability at production and consumption was consequently highlighted in some PIMENTO reviews, particularly where live microorganisms were considered part of the active exposure (44, 49, 54, 61).

The PIMENTO reviews have shown that fermentation can synthesise bioactive compounds and/or increase their concentration in the food matrix, and that some of these compounds are storage labile. In human studies focusing on vitamins, menaquinones (K2) in natto and certain cheeses appear to be present in sufficient concentrations to improve K-status in humans, whereas naturally occurring folates in FF were noted to be chemically labile and prone to loss during storage (59). In whey-based products, peptides and organic acids were retained even when the products were pasteurised or dried, despite the absence of microbial viability (56). Few human studies reviewed by PIMENTO have quantified target bioactive levels in foods at delivery; therefore, the time-dependent losses (or stability) of specific compounds generally remain unverified (47, 61).

##### 2.3.3.5 Analytical tools to characterize food compounds

Several PIMENTO reviews have reported the use of modern analytical toolkits to characterise FF in the context of human trials. Microbiological approaches ranged from traditional plate counts of viable LAB and yeasts to DNA-based methods, such as 16S rRNA sequencing, qPCR, and metagenomics, with a few studies specifying strain-level identification where claims depended on particular microbes (44, 47, 61). From a chemical and nutritional perspective, HPLC and UPLC-MS have been applied to quantify vitamins (K2 and B-vitamins), polyphenols, organic acids, amino acids, and peptides, whereas GC has been used to quantify volatiles and short-chain fatty acids (61). Stable isotope dilution was employed in vitamin studies, for example to improve the quantitative reliability of folate analysis (59). Untargeted metabolomics was highlighted as a method for capturing a broader profile of fermentation-derived metabolites (47, 61). Finally, tools to assess bioavailability and mechanisms included stable isotope tracers to compare absorption from fermented versus non-fermented matrices and mechanistic biomarkers such as cytokine measurements to test anti-inflammatory effects (43). Nikolaou et al. (55) reported the use of tracer based approaches, including stable isotope-based approaches, to assess iron absorption in relation to fermentation associated phytate reduction, although the overall human evidence remained context dependent and inconclusive.

##### 2.3.3.6 Consistency in analytical characterisation of fermented foods

Across PIMENTO reviews, the analytical rigour for product characterisation was reported as variable. For example, Humblot et al. (48) noted that only a small number of intervention studies identified microorganisms at the genus or strain level, with strain identification completed only at a generic level, despite strain-specific functionality being critical to FF characteristics (47, 61). Künili et al. additionally reported that only a minority of trials quantified the hypothesised bioactives in foods or participants (61). Many studies inferred composition from prior literature (e.g., natto → K2; kimchi → peptides) without verifying their assumption in the study batch. A few studies provided more complete characterisation, including named strains and viable counts, such as fermented whey products containing *Lacticaseibacillus rhamnosus* GG at 10^8^ CFU/mL (56).

#### 2.3.4 Safety

Across the body of evidence collected in the PIMENTO reviews, safety reporting for FF interventions is generally limited; however, where assessed, outcomes are consistently reassuring (47). Adverse events are usually either not reported or explicitly stated as absent in several studies. When reported, adverse events are typically mild, transient, and most often gastrointestinal in nature (e.g., diarrhoea or bloating); these often overlap with the outcomes under investigation themselves (42, 44, 57). More significant effects are rarely noted; for instance, a single trial reported discontinuations due to severe bloating, while non-gastrointestinal symptoms were uncommon. The tolerability profile of FF was generally reported favourably among PIMENTO reviews, supported by the long-standing safe use of LAB and other fermentative microorganisms in food systems and processes. This is reflected in their regulatory status (e.g., GRAS/QPS), although several PIMENTO reviews cautioned that such assumptions often replaced systematic safety assessment in primary studies, leading to underreporting or incomplete evaluation of adverse events (48, 49). Consequently, even where no safety concerns were identified, the lack of comprehensive monitoring and consistent reporting limits the strength of safety conclusions. The absence of reported adverse events in a given study should not, on its own, be interpreted as evidence of safety: it reflects inconsistent and often incomplete adverse-event reporting across the included reviews rather than a demonstrated absence of risk. The discussion above concerns clinical tolerability, i.e., patient-reported symptom outcomes in human intervention studies. The following observations instead concern microbiological and chemical safety, i.e., contamination and hazard exposure, addressed separately and in greater depth in the WG3 Safety review referenced in the Methods (Section 2.2.1). Importantly, in studies where mechanistic or functional considerations were explored, fermentation itself was often associated with potential safety advantages. These included reductions in intestinal inflammation or permeability in human studies, degradation of immunogenic compounds such as prolamins through specific microbial activity, and technological benefits such as pH reduction and organic acid production that enhance microbiological safety and may reduce chemical hazards like acrylamide precursors (43, 45). At the same time, authors noted that these benefits depend on appropriate process control, as inadequate fermentation conditions could permit accumulation of undesirable compounds (e.g., biogenic amines) or microbial risks. Finally, some of the narrative and scoping PIMENTO reviews provided only minimal or implicit consideration of safety, often noting that it was not systematically assessed or could not be reliably inferred from observational designs (59). This reinforces a broader methodological limitation in the literature: safety is frequently assumed rather than rigorously evaluated.

When examining observations related to specific FF product categories, findings were consistent with the overall patterns described above. Fermented cereal products such as sourdough bread were not associated with major safety concerns; occasional mild gastrointestinal complaints were more plausibly linked to ingredients rather than fermentation itself, and intrinsic processing steps (e.g., baking, low water activity) contributed to low microbiological risk (45, 55). Similarly, fermented dairy products were generally well tolerated across diverse adult populations, although subgroup-specific risks remain insufficiently studied (47). Fermented wheat germ extract and whey-based fermented products also demonstrated good clinical tolerability, including the absence of hepatic or renal safety signals, while highlighting the need for improved standardisation and characterisation of production processes (56, 58). One dedicated PIMENTO review on FF safety by Assuncao et al. (41) reports on chemical and microbiological hazards across commodities and geographies. Cheese, yogurt, and fermented meats are among the fermented foods most frequently implicated in foodborne outbreaks of microbiological origin, most likely because these are some of the most frequently consumed FF in Europe (4, 101, 102) and in the US (103).

Despite this broadly positive safety profile, several important caveats were identified. Evidence remains limited and heterogeneous in certain populations, including individuals with allergies, lactose intolerance, irritable bowel syndrome, or compromised immune function, for whom caution is still advised. In addition, many trials excluded such groups altogether, further restricting generalizability (44, 56). Some PIMENTO reviews also highlighted the importance of strain-level characterisation as a prerequisite for substantiating both probiotic claims and safety assurance (47, 49).

When evaluated collectively, the safety profile of fermented foods was consistently unremarkable: no consistent serious adverse signals were identified in the populations studied, indicating a favourable safety and tolerability profile for FF. This cumulative impression aligns with broader state-of-the-art summaries on FF, which describe a long history of safe use when products are prepared under hygienic conditions and with appropriate process control (50). Most hazards appear to originate upstream, from contamination of raw materials and the primary production environment (such as lapses in hygiene or temperature control before or during fermentation), or from the production of a small set of predictable process-related chemicals (particularly biogenic amines) rather than being intrinsically generated by the fermentation step when conducted with good hygiene and process control. This interpretation is consistent with domain reviews and public-health guidance, which repeatedly trace hazard entry points to contaminated cereals, legumes, fruits, fish, milk, or spices, as well as to poor handling before fermentation (104). From a public-health and regulatory perspective, a pragmatic and proportionate approach is warranted with continued surveillance, transparent, consistent and standardised reporting of adverse events, and integration of safety assessment within efficacy trials to maintain consumer confidence and support responsible innovation.

### 2.4 Totality of evidence and knowledge gaps for EFSA health claim requirements

The common structure of the PIMENTO reviews in relation to EFSA health claim requirements aimed at evaluating the collected evidence as a whole (35). This evaluation provided well substantiated insights into the current levels of available evidence for FF in relation to health, while highlighting the potential knowledge gaps. Across the spectrum of fermented foods and outcomes evaluated in the PIMENTO reviews, a convergent inference was the need to further investigate the potential beneficial role of FF on health. Although current evidence suggests a positive trend, it lacks the objective, structured, and consistent data often required for regulatory approvals. This is partly due to the unique and complex nature of FF, which can make designing clinical trials or interpreting observational studies particularly a challenge, as observed across the diverse human studies evaluated in the PIMENTO reviews. Below, we briefly discuss totality of the evidence gathered in the PIMENTO reviews, expounding on the general observations mentioned above, in terms of the potential requirements for an EFSA (or other similar regulatory body) health claim dossier, as well as the knowledge gaps that currently exist in this area as informed by these observations.

#### 2.4.1 Characterisation of fermented foods

Characterisation of the food or food constituent with a potential health or nutritional effect is a cornerstone of the EFSA health claim dossier. Details of FF studied, such as quantity ingested, composition, origin and composition of raw materials, process used, microorganisms present, and description of the food matrix, are almost always inadequately described, often heterogeneous and generally incomplete. This lack of detail hinders a robust causal interpretation of health effects (48).

##### 2.4.1.1 Raw materials and composition

The FF examined across PIMENTO reviews covered a broad range of raw materials and products, including dairy, cereals, legumes, soy, fish, meat, fruits, vegetables, and derived products such as fermented whey and fermented wheat germ extract. However, the evidence base was dominated by dairy products, whereas non-dairy, traditional, artisanal, and region-specific FF were underrepresented. In many studies, raw material characterisation was limited to broad food categories or basic nutritional descriptors, with only limited reporting of fibre, micronutrients, or other relevant compounds (42, 44, 45, 47, 48, 59, 61). This is important because specific raw material-microbe pairings can influence nutritional and functional properties, as illustrated by natto as a source of menaquinone-7, milk fermented with *Propionibacterium* spp. as a source of vitamins B12 and K2, and cereal fermentations associated with higher B-vitamin levels (59). Although specific raw material-microbe combinations can generate distinct bioactive profiles, the lack of detailed and standardised reporting on both inputs and fermentation processes limits interpretability, comparability, and accurate assessment of dietary exposure (42, 61).

##### 2.4.1.2 Manufacturing process

Fermentation processes are also described inconsistently. Key parameters, including microbial composition, starter cultures, fermentation type and duration, temperature, pH, oxygen conditions, and downstream processing, are often missing or insufficiently detailed (42–45, 47–49, 56, 57, 59, 61). This is not a minor technical issue, as these parameters directly influence the composition and functionality of the final product. Some studies provided partial information, particularly in cereal or whey-based products, but even there, downstream processing and post-fermentation handling were not consistently documented. In other cases, foods such as yogurt, kefir, kimchi, or fermented meats were named without sufficient detail on how they had been produced, whether they had undergone heat treatment, or how far traditional and industrial processes differed (44, 46, 54, 61). While some studies provided partial details (e.g., fermented cereals reporting fermentation time and temperature, or whey products describing substrates and cultures), downstream processing steps remained inconsistently documented (45, 47, 56). Overall, inconsistent and incomplete process characterisation constrains reproducibility, comparability, and regulatory interpretation, particularly given that these parameters directly influence the composition and functionality of the final product; therefore, standardised and detailed reporting of fermentation conditions and processing is essential for both scientific validity and regulatory assessment.

##### 2.4.1.3 Composition of end products

Across PIMENTO reviews, end-product characterisation is consistently incomplete, limiting both the interpretability and comparability of findings. This restricted comparability across studies and interpretation of the biological signals attributed to fermentation. In yogurt-based interventions, viable lactobacilli were often considered the active component, yet strain identity and stability within the food matrix were seldom verified. Product labels were commonly used instead of molecular confirmation, with only isolated exceptions (44, 49). In cereal-based studies, changes in fibre fractions and phenolics were sometimes reported, but microbial content, fermentation-derived metabolites, and structural properties of fermented breads were rarely characterised in depth (45, 61). Similar limitations were evident in vitamin-focused studies, where the contribution of microbial synthesis to final vitamin content was often unclear, and in FWGE, where selected benzoquinones were identified but broader compositional profiling remained incomplete (58, 59). Across domains, studies also rarely quantified the bioactive compounds hypothesised to mediate health effects, so exposure was often inferred rather than directly measured. Overall, insufficient profiling of nutrients, microbes, and key metabolites remains a major limitation in the current human literature.

##### 2.4.1.4 Stability and storage

Shelf life, storage conditions, batch-to-batch variability, and post-fermentation handling are generally not assessed or reported, limiting assessment of exposure reliability and product comparability. Similarly, storage-related changes in vitamins, peptides, organic acids, or other bioactive compounds were seldom quantified. These omissions limit confidence in the actual exposure delivered to participants and further reduce comparability across studies.

##### 2.4.1.5 Quality of analytical tools

The analytical rigour used to characterise FF is highly variable. Observational studies frequently relied on self-reported intake tools, increasing the risk of exposure misclassification (42, 44, 46–48, 54). In intervention studies, methods used to identify and quantify microbes, metabolites, and bioactive compounds were inconsistently reported and rarely harmonised across projects (42, 44, 47, 48, 57, 59, 61). Although modern analytical approaches were sometimes cited, validation and reporting of results were often incomplete, and product composition was sometimes inferred from prior literature or product labels rather than verified in the actual intervention batch. This limited the ability to distinguish effects attributable to fermentation from those attributable to the base matrix and constrained the reproducibility and regulatory interpretability of the evidence.

##### 2.4.1.6 Overall conclusion

The composition of fermented foods is often reported superficially or inadequately with the characterisation of FF described as “neither convincing nor sufficient” across projects for regulatory substantiation, though it may be adequate for hypothesis generation. This is a significant limitation because the food matrix itself can exert strong biological effects, making it difficult to isolate any additional contribution of fermentation when the product is poorly defined (42, 44). Currently, the available evidence does not support treating of fermentation as a predictable, standalone causal factor across FF. More detailed and standardised characterisation of raw materials, production processes, end-product composition, stability, and analytical verification will therefore be essential for both scientific interpretation and regulatory evaluation.

#### 2.4.2 Relationship between consumption of fermented foods and functional effects

##### 2.4.2.1 Specificity of effect

Specificity of effects varied by health outcome and FF category, but overall attribution is difficult. Several PIMENTO reviews (Table 1) reported associations, with variable consistency, across a broad spectrum of outcomes, including cardiometabolic health, antioxidant status, immune function, gastrointestinal health, cognition, bone health, developmental outcomes, cancer-related endpoints, and mortality (46–49, 51, 54, 56–58, 61), where specificity of effect was limited, as similar outcomes were linked to multiple FF categories and bioactive classes. More consistent effects were reported in reviews where the intervention or exposure was explicitly linked to microbial features, such as fermented foods containing probiotic bacteria for vaginal health or foods fermented with *Lactobacillus* and/or *Bifidobacterium* species for cognitive health (47, 49), This does not imply that other outcomes are independent of microbial activity. As FF often contains probiotic strains, understanding the mechanisms of action contributing to the potential health effects of these food products requests that appropriate controls be conducted that separate the ability of these strains to ferment the food matrix from their probiotic activity in the gastrointestinal tract. Cardiometabolic, glycaemic, gastrointestinal, bone, and lipid outcomes remain biologically plausible but incompletely demonstrated in human studies, and the reported effects were generally modest, variable, or inconsistent (42, 44, 45, 57). In food allergy studies, fermentation sometimes reduced allergenicity but could also generate novel allergens, limiting specificity (43).

##### 2.4.2.2 Dose–response relationship and lowest effective dose

Dose–response relationships were rarely assessed and were not established in any of the PIMENTO reviews. Most intervention studies used fixed doses, and only isolated studies explored more than one intake level (42, 44, 45, 47, 49, 57). Although some studies quantified intakes of peptides, polyphenols, or GABA, systematic evaluation of dose thresholds was lacking (56, 58, 61). Consequently, the lowest effective dose remains undefined for all outcomes studied across the PIMENTO reviews.

##### 2.4.2.3 Magnitude and physiological relevance

Underlying review-level findings, including effect estimates and outcome detail where reported by the source reviews, are tabulated in the “Main Results” column of Supplementary Table S1. Observed effects were generally small to modest. Some findings were potentially physiologically relevant, including reduced bacterial vaginosis incidence, modest LDL-c reductions, slight attenuation of postprandial glycemia, and cognitive improvements in older adults, but effect sizes were often insufficient to increase certainty or meet clinically-relevant thresholds (42, 44, 45, 47, 49, 57). In bone health, effects on BMD were minimal and below clinical relevance (44). Findings on fermented wheat germ extract suggested possible therapeutic relevance, particularly in adjunctive cancer settings, but evidence remained inconsistent and based on small set of studies and study participants (58).

##### 2.4.2.4 Consistency across studies

Consistency across studies was limited. This reflected heterogeneity in FF type, microbial composition, study design, intervention duration, populations, and outcome measures (42–45, 47–49, 56, 57, 61). Observational studies sometimes showed more consistent long-term associations, such as for mortality or cognition, but these did not allow causal inference (42, 44, 47). Cohort studies in some PIMENTO reviews showed relatively consistent associations between fermented dairy consumption and reduced all-cause and CVD mortality (46, 54), while evidence for other FF categories was mixed or sparse.

##### 2.4.2.5 Representativity of study groups

Study populations were generally relevant to the outcomes under investigation, including healthy adults, older adults, women, and mildly at-risk groups. However, vulnerable or deficient subgroups were underrepresented in several projects (43, 44). In other PIMENTO reviews, the populations studied ranged more widely, from infants to older adults and from healthy individuals to patients with chronic disease (46, 51, 54, 58). Many studies, however, were based on small and relatively homogeneous groups, which limits generalizability. Cross-country comparisons were also complicated by population-specific consumption patterns (48).

##### 2.4.2.6 Conditions under which effects were achieved

Effects were observed under highly variable conditions, including free-living habitual dietary intake, controlled feeding, short-term postprandial tests, adjunctive clinical treatments, and other clinical settings. The lack of harmonisation in exposure assessment and outcome measurement limits comparability and therefore the strength of the evidence for the investigated health effects (42–45, 47–49, 56, 57, 61).

##### 2.4.2.7 Sustainability of effects

Long-term sustainability could not be assessed due to short intervention durations and lack of follow-up (42–45, 47, 49, 56–58, 61). Observational data suggested possible sustained associations for some outcomes, including mortality, but these findings do not establish causality (42, 44, 46, 47, 54).

##### 2.4.2.8 Reasonable amount of fermented food consumption

The amounts consumed in most studies were generally compatible with habitual intake, such as one to two servings per day of yogurt or fermented milk, or 88–200 g per day in studies of bacterial vaginosis (42, 44, 46, 47, 49, 54). For specific products (FWGE, fermented whey), intake levels were study-specific and not optimised (56, 58). Overall, realistic intake ranges were often used, but optimal amounts remain undefined. Defining optimal or minimal effective intake levels will require dose–response studies that relate clearly characterised FF exposures to validated clinical, nutritional, or mechanistic endpoints.

##### 2.4.2.9 Overall conclusion

Across the PIMENTO reviews, the evidence for functional effects of FF consumption was neither convincing nor sufficient for regulatory substantiation. Although several projects suggested possible benefits or associations, causal inference remained limited by heterogeneity, imprecision, modest effect sizes, lack of dose–response data, and limited information on long-term sustainability (41–49, 51, 54–59, 61).

#### 2.4.3 Supportive evidence

##### 2.4.3.1 Bioavailability

The PIMENTO reviews report that fermentation was frequently discussed as a process that may improve bioavailability, increase bioaccessibility, or alter the kinetics of nutrient and metabolite appearance in peripheral blood by modifying the food matrix, reducing antinutritional factors, and generating or releasing compounds such as peptides, amino acids, organic acids, vitamins, polyphenols, GABA, short-chain fatty acids, and other microbial metabolites (42, 44–47, 51, 54, 56–59, 61). Illustrative examples included improved calcium uptake from yogurt and the conversion of isoflavone glycosides into more bioavailable aglycones. However, direct human evidence remains limited. Only a minority of studies quantified the target bioactive compound before and after fermentation, measured systemic exposure to relevant metabolites, or assessed microbial survival *in vivo*. Likewise, improvements in acute bioavailability did not necessarily translate into sustained improvements in nutritional or functional status. Key determinants such as microbial strain, fermentation conditions, food matrix, and inter-individual variability were also rarely characterised in sufficient detail. As a result, the biological plausibility of improved bioavailability is stronger than the direct human evidence currently available.

##### 2.4.3.2 Mechanism of action

Mechanistic evidence across the PIMENTO reviews was largely indirect and derived predominantly from preclinical studies or inference rather than direct validation in humans. Proposed mechanisms included modulation of gut microbiota and barrier function, pathogen exclusion, immune and inflammatory regulation, microbial synthesis of vitamins and bioactive peptides, production of short-chain fatty acids and neuroactive compounds, and signalling along the microbiota-gut-brain axis (46–49, 51, 54, 56, 58, 59, 61). Additional hypotheses involved fermentation-induced structural changes and organic acid production that may slow digestion and influence glycaemic or lipid responses, as well as pathways relevant to neuroinflammation, neurotransmitter synthesis, and BDNF signalling in cognitive contexts (42, 47). This is particularly relevant for dairy products, where fermentation can alter the protein matrix and may modify amino-acid appearance kinetics compared with milk, as illustrated by yoghurt (76). However, human trials were rarely designed to test these pathways directly, with limited use of mechanistic biomarkers, sparse strain-specific investigation, and little pathway-level confirmation. Consequently, many mechanisms proposed across the studies reviewed by PIMENTO remain plausible but insufficiently demonstrated in humans. This gap between biochemical plausibility and empirical confirmation is one of the main reasons why claims related to FF remain insufficiently substantiated from a regulatory perspective (42, 44, 45, 47).

##### 2.4.3.3 Overall conclusion

Supportive evidence on bioavailability and mechanisms is biologically plausible, but remains insufficiently demonstrated in humans, and does not substantiate causal claims.

#### 2.4.4 Safety

Although safety is not an evidential criterion for EFSA health claim substantiation, it is an important part of the evaluation of the health benefits and risks of fermented foods and an important part of any clinical trial investigating FF. The PIMENTO reviews report that the FF studied were generally well tolerated in the populations investigated, with no major safety concerns reported in most intervention settings, as detailed in the Safety section above. This may partly reflect the fact that many fermented foods are widely consumed as part of habitual diets and have a long history of dietary use. However, absence of major safety signals in these studies should not be interpreted as definitive evidence of safety across all products, processes, doses, or populations, because safety monitoring was often limited and vulnerable groups were frequently underrepresented. Overall, the main safety concerns identified for FF were primarily linked to upstream contamination of raw materials, the production environment, or the formation of undesirable compounds under poor hygiene or inadequate process control, rather than to fermentation itself.

#### 2.4.5 Knowledge gaps

Knowledge gaps identified from the total evidence and observations across all PIMENTO reviews are briefly discussed below. The knowledge gaps were categorised into nine primary domains with a priority designation in parentheses: (i) *HIGH* (gaps that currently *block* scientific progress, mechanistic understanding, or regulatory substantiation), (ii) *MEDIUM* (important gaps that improve evidence quality but do not fully block progress), and, (iii) *LOW* (valuable strategic needs, but not essential for immediate advancement). An overview of the knowledge gaps and how they link with various roadmap actions (see section below) is presented in Figure 1 and Supplementary Table S2.

##### 2.4.5.1 Food characterisation and composition

- *Lack of diversity in fermented foods studied (HIGH).* Current evidence is dominated by dairy-based FF, limiting cultural/general applicability. Traditional and artisanal fermented foods remain poorly characterised, restricting our understanding of global FF health potential.
- *Incomplete characterisation of raw materials (HIGH).* Missing micronutrient, fibre, and bioactive data obscures which components, not fermentation *per se*, drive effects. This currently limits mechanistic and clinical interpretation.
- *Limited understanding of substrate–microbe interactions (MEDIUM–HIGH).* Specific substrate-microbe combinations determine bioactive formation, but these interactions are rarely studied. Without them, predictive modelling of FF functionality is impossible.
- *Lack of comprehensive end product composition profiling (HIGH).* Missing microbial identity (particularly at the strain level), viability, metabolite profiles, and matrix properties in the end fermented products prevents comparison across studies and blocks regulatory substantiation. A minimum reporting checklist for fermented food characterisation would directly address this gap; this can potentially act as a proxy measurable outcome for progress on the proposed roadmap.
- *Poor documentation of intracategory variability (MEDIUM).* Batch variation and regional manufacturing differences are almost never recorded. This creates ambiguity in intervention reproducibility.

##### 2.4.5.2 Fermentation process and manufacturing

- *Inadequate reporting of fermentation parameters (HIGH).* Critical parameters (time, temperature, pH, oxygen, strain specificity) are missing in most studies, making them scientifically non-reproducible and non-regulatory-grade.
- *No harmonised fermentation reporting standards (HIGH)*. The absence of a unified reporting framework is a major structural barrier to progress. A CONSORT-style guide for FF is urgently needed.
- *Limited linkage between process → composition → outcomes (HIGH).* Very few studies examine all three layers in an integrated manner, preventing causal inference about what aspects of fermentation drive health effects.
- *Proprietary constraints limit transparency (LOW-MEDIUM).* Some processes (e.g., fermented wheat germ extract) are not publicly described. This hampers independent scientific evaluation.

##### 2.4.5.3 Stability, storage and exposure

- *Lack of microbe viability/metabolite stability data over shelf life (HIGH).* Exposure estimates cannot be trusted without time-resolved viability and metabolite retention data from production all the way to consumption.
- *Poor estimation of consumed dose (HIGH).* Many studies measure composition at production but not at intake, fundamentally compromising exposure classification.
- *Limited data on storage impacts on safety and efficacy (MEDIUM).* Storage-driven changes in microbial activity or metabolite degradation are rarely measured, although these may influence tolerability or risk.

##### 2.4.5.4 Analytical methods

- *Lack of harmonised, validated analytical frameworks (HIGH)*. Analytical inconsistency is one of the most pervasive barriers to synthesis, comparability, and regulatory acceptance.
- *Analytical measures not aligned with mechanistic hypotheses (MEDIUM)*. Bioactive compounds are often not pre-specified or measured, weakening mechanistic interpretation and trial design efficiency.
- *No fermented-food composition databases (MEDIUM-LOW)*. A long-term but valuable goal that would support epidemiology, but not essential for immediate intervention research. The establishment and growth of such a database, eventually linking fermented food characteristics with preclinical and clinical outcome data, can potentially act as a proxy measurable outcome for progress on the proposed roadmap.

##### 2.4.5.5 Human evidence and study design

- *Poor specificity of effect across FF (HIGH).* Multiple FF map to similar outcomes, making attribution impossible under current evidence structures. This is a critical regulatory limitation.
- *Lack of dose–response evidence (HIGH).* No FF category has defined thresholds or minimal effective doses.
- *Effect sizes are small/modest (MEDIUM).* Most effects fall below clinical relevance thresholds, though better-designed studies may clarify this. Urgency is moderate.
- *Extremely high heterogeneity across studies (HIGH).* Differences in FF types, strains, methods, outcomes, and durations severely limit inference. Addressing this is foundational for any synthesis effort.
- *Limited longitudinal and follow-up data (MEDIUM).* Long term data from longitudinal studies and follow-up studies are scarce. This is essential for chronic-disease outcomes.
- *Poor comparators (HIGH).* Use of mismatched comparators prevents attribution of effects to *fermentation itself*. Fixing this is fundamental for causal inference. A standardised, harmonised guideline for fermented food trial conduct would directly address this gap; this can potentially act as a proxy measurable outcome for progress on the proposed roadmap.
- *Underrepresentation of vulnerable populations (MEDIUM).* Important for population relevance but secondary to analytical and process-level deficiencies.
- *Habitual FF intake rarely assessed (MEDIUM-LOW).* This gap affects interpretation but is not the single most urgent step.

##### 2.4.5.6 Mechanisms of action and bioavailability

- *Lack of human evidence for enhanced bioavailability (HIGH).* Bioavailability claims remain largely preclinical, leaving a major gap between mechanistic plausibility and human confirmation.
- *Mechanistic human studies lack biomarkers or pathway validation (HIGH).* Without biomarker-integrated designs, mechanistic confidence remains weak, directly blocking regulatory substantiation.
- *Lack of linkages between bioactive levels and physiological effects (HIGH).* Studies are often carried out in *in vitro* or preclinical setups, with changes in bioactive concentrations in FF due to fermentation never linked to actual, physiological levels and/or effects in humans.
- *Determinants of bioavailability not studied together (MEDIUM).* Food matrix, vitamers, fermentation conditions, and host microbiota are rarely integrated; essential but not the single most urgent step.
- *Mechanistic evidence is insufficient for regulatory requirements (HIGH).* Mechanistic evidence is almost always absent for studies or presupposed. This is one of the primary reasons EFSA repeatedly concludes evidence as “neither convincing nor sufficient.”

##### 2.4.5.7 Vitamins and minerals

- *Limited human studies linking FF-derived vitamins to status (HIGH).* Especially regarding vitamin B12 and non-K, non-folate vitamins. This is a key missed public-health opportunity.
- *Limited human evidence linking fermentation-mediated mineral bioavailability to mineral status* (HIGH). Sourdough and other cereal fermentations may reduce phytate and thereby plausibly improve iron bioavailability, but direct human evidence demonstrating improved iron absorption or iron status remains insufficient.
- *Poorly characterised mechanisms of vitamin production (MEDIUM).* Strains, vitamers, and fermentation conditions remain insufficiently studied. These factors are important for optimization but not immediate.
- *FF-derived vitamins and fermentation-mediated changes in mineral bioavailability missing from dietary assessments (LOW-MEDIUM).* Long-term goal needed for better epidemiology but less urgent than mechanistic or intervention-level gaps.

##### 2.4.5.8 Dietary intake assessment

- *High misclassification from FFQ/recall (HIGH).* Dietary misclassification undermines all observational results; correcting this is central to epidemiological validity.
- *Participants may not recognise FF (MEDIUM).* An important but secondary contributor to exposure misclassification.
- *Poor differentiation of FF subcategories in surveys (HIGH).* Broad categories (e.g., “fermented dairy”) mask critical biological differences, leading to misleading associations.

##### 2.4.5.9 Safety

- *Inconsistent safety reporting in trials (HIGH).* Systematic safety endpoints and adverse-event tracking are rare, leaving an incomplete picture of tolerability.
- *Limited safety data in vulnerable groups (HIGH).* Safety assessment is particularly important in vulnerable populations, including infants, older adults, pregnant individuals, immunocompromised people, and individuals with allergies, gastrointestinal disorders, or metabolic disease.
- *Clinical studies overlook real-world contamination risks (LOW-MEDIUM).* Food-borne hazards are well documented yet rarely incorporated into FF trials; valuable but not a blocker for efficacy research.

#### 2.4.6 Integrated conclusion on totality of evidence

Of the 13 PIMENTO reviews assessed against EFSA’s evidentiary categories (Table 1), none reached the threshold of convincing and sufficient evidence; although these reviews clearly identified evidence for the health properties of FF, the totality of evidence is accordingly assessed as neither convincing nor sufficient. When evaluated collectively and organised according to EFSA evidence requirements and beyond, the totality of evidence derived from the PIMENTO reviews indicates that *“the evidence for a cause-effect relationship between consumption of FF and the claimed health effects is NEITHER CONVINCING NOR SUFFICIENT.”* This conclusion reflects several recurring limitations, including insufficient food characterisation, lack of dose–response data, modest and inconsistent effects, and limited evidence on bioavailability and mechanisms of action. Progress in this field will therefore depend on substantially greater rigour in human intervention studies, especially RCTs. Future studies should use well-characterised FF interventions, appropriate comparators selected according to the research question, adequate intervention duration, predefined dose–response designs where feasible, and mechanistic or bioavailability endpoints aligned with the hypothesised effect. Comparator selection is particularly important because many existing studies do not allow the effect of fermentation to be separated from the effect of the raw food matrix, added probiotic strains, or other interventions. We emphasise, however, that this evaluation states that there is evidence. Taken together, the series of PIMENTO reviews provide supportive and hypothesis-generating evidence that points to biological plausibility and potential health relevance, but not to a level that would currently support regulatory substantiation of causal claims. The knowledge gaps identified in this section help define priorities for future research and provide the basis for the strategic roadmap presented below (Figure 1; Supplementary Table S2).

## 3 Strategic roadmap

The regulatory “gold standard” for validating health effects remains the authorisation of health claims by national or international authorities (e.g., EFSA in Europe, FDA in the United States) following independent scientific evaluation. To date, only two examples related to FF stand out, both for yogurt (30, 31). An EFSA-authorised health claim under Regulation (EC) No 1924/2006, and an FDA qualified health claim, a lower evidentiary tier. Of note, the FDA has qualified the health claim on yoghurt and diabetes as “limited,” to a large extent because the controls used for the intervention studies included into the dossier being inappropriate to address the research question (31). These cases are nonetheless highly instructive as they show how fermented foods, standardised in composition (nutritional, metabolic, and microbiological) using appropriate comparators and complete elucidation of the mechanism of action, along with a long history of safe consumption (presumed safety), among other factors, can provide a blueprint for strengthening the evidence for the health properties of FF. More broadly, progressing from fragmented signals to convincing substantiation requires coordinated effort across fermented-food characterisation, human evidence, and mechanistic validation (Figure 2). Most FF are not as well characterised or standardised in their production, stability, and bioactivity; combined with poor experimental designs and hypothetical mechanisms of action, the available data fails to demonstrate a clear cause-effect relationship, resulting in limited scientific evidence for their health properties. The next two sections discuss, as informed by the knowledge gaps presented above, how scientific research and policy should address these issues in time with the long-term goal of defining recommended intake levels for specific fermented foods categories, health outcomes, and populations, rather than for FF as an undifferentiated group, and their inclusion in dietary guidelines (Figure 2; Supplementary Table S2). It must be noted that, the PIMENTO WG3 initiative and Strategic Roadmap, is indeed guided by scientific workflows proposed by regulatory authorities, particularly the EFSA, and utilised the methodological framework laid out by such regulatory offices to harmonise the research carried out during WG3 implementation. However, while the PIMENTO WG3 initiative recognises that regulatory and approval oriented scientific workflows can significantly *strengthen the evidence* for the health properties of fermented foods and accelerate translation into dietary practice, it does not propose that the primary focus of FF research should revolve around possible functional approvals; the following Roadmap is hence targeted at diverse stakeholders with an aim to provide a holistic and comprehensive outlook for FF in the near future. For clarity of presentation, the objectives below are organised into Research and Policy sections; however, it is to be noted that, these represent two facets of a single, integrated Strategic Roadmap rather than two independent roadmaps. The priority levels and time horizons that follow represent provisional expert judgements of the author panel, not the output of a formal prioritisation procedure, and should be read and used accordingly. For the Roadmaps, we have classified our proposed objectives into short- (0–3 years), medium- (3–7 years), and long-term (7–15 + years) categories, largely informed by the priority levels mentioned in the Knowledge Gaps section above. However, it is important to recognise that while an objective may be classified as a short-term goal based on priority, full realisation of the objective may take years and may continue beyond the 15 year-timeline assigned here.

**Figure 2:**
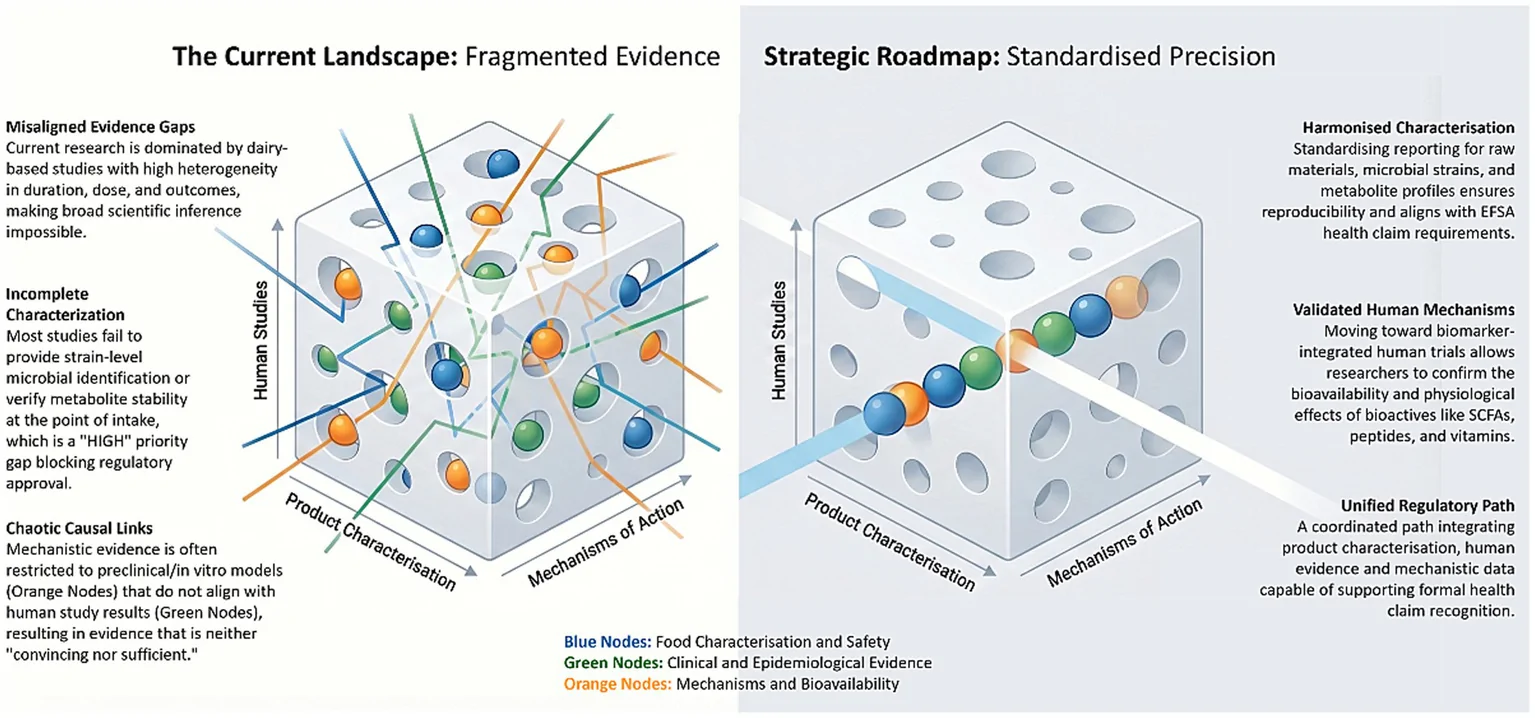
Conceptual framework of research strategy to demonstrate the health properties of fermented foods: Pushing light through the “Swiss cheese” of fragmented evidence. In analogy to the scattered holes in Swiss cheese, current research on the health properties of fermented foods is distributed across a large, heterogeneous body of uncoordinated human trials, incomplete food characterisations, and isolated mechanistic data. This fragmentation prevents an objective evaluation of the totality of the evidence, often resulting in regulatory assessments that deem the data neither convincing nor sufficient. The three axes in the Figure represent the critical domains required for regulatory-grade science: Comprehensive product characterisation (including strain identity and bioactive stability), Rigorous human clinical studies (well-powered randomised controlled trials), and Validated mechanisms of action (bioavailability and human biomarkers). To transform diffuse, weak evidence into a robust scientific consensus, research resources (the “light beam”) must be aggressively coordinated and focused. By aligning specific, promising combinations of fermented foods with targeted clinical indications across all three axes simultaneously, the field can push through the fragmented landscape to produce sufficient, convincing, and regulatory-grade evidence.

### 3.1 Research roadmap

#### 3.1.1 Short-term research roadmap: standardisation and foundational tools

In the short term, priority should be given to standardisation of various aspects of FF studies to establish a comprehensive foundation to be set for further research in the coming decades. To this end, one of the primary areas of concern that requires resolution is harmonisation of FF characterisation at a depth sufficient for reproducible results and regulatory approval. Indeed, across the PIMENTO reviews, one of the most persistent limitations observed in the examined studies was insufficient reporting of raw materials, fermentation conditions, microbial composition, post-fermentation processing, viability at consumption, and the concentration or stability of hypothesised bioactive compounds, among other factors. This lack of detail prevents meaningful comparison across studies and weakens causal inference. Future intervention studies should therefore adopt minimum reporting standards aligned with EFSA requirements, covering raw materials, process parameters, microbial information at the strain level where relevant, metabolite profiles, final product composition, and stability at the point of consumption. Standardised product dossiers would further improve transparency, reproducibility, and scientific relevance.

A second short-term priority for improvement in standardisation is exposure assessment. Observational studies encountered in PIMENTO reviews showed that interpretation remains difficult because FF are often grouped into broad and biologically uninformative categories, with dietary instruments rarely distinguishing products that differ in matrix, process, microbial content, or metabolite profile. Fermented food frequency questionnaires (3FQ) (101, 105) should therefore be further validated and more widely used, including AI-based dietary assessments, and the classification of FF in cohort studies and dietary surveys should be refined beyond broad categories such as fermented dairy. Where feasible, exposure assessment should also move toward quantifying microbial exposure from foods, including both living and nonviable microorganisms consumed. Without more precise exposure assessment, observational evidence will continue to suffer from misclassification and will remain of limited value to strengthen the evidence for the health properties of FF.

Short-term progress also depends on stronger analytical and methodological rigour. Analytical methods for microbes, metabolites, vitamins, and other putative bioactive compounds should be better standardised, and studies should quantify the compounds hypothesised to mediate the reported effects rather than infer composition from previous literature. Greater attention should also be paid to traceability, batch consistency, and reproducibility. Study design must be strengthened using appropriate comparators, including non-fermented matrices, pasteurised fermented products, or matched composition, depending on the research question. Intervention studies should explicitly state whether a meaningful non-fermented comparator exists, as this directly affects how confidently the effect of fermentation can be separated from that of the starting raw material. Human trials should also report dose, duration, and intervention composition more consistently and be designed around a clear mechanistic hypothesis rather than vaguely defined FF exposures.

#### 3.1.2 Medium-term research roadmap: mechanistic and translational research

In the medium-term, research on FF should gradually shift toward understanding mechanistic aspects and translational validation in humans. Many proposed mechanisms for FF are biologically plausible, but they remain supported mainly by preclinical or indirect evidence. Future studies should therefore include biomarker driven designs capable of testing the bioavailability of fermentation derived compounds, the survival or functional activity of relevant microorganisms, pharmacokinetic characteristics of the bioactive compounds, and the physiological relevance of hypothesised pathways *in vivo*. This is especially important for peptides, vitamins, phenolic metabolites, GABA, and other bioactive compounds, for which health effects are often proposed without direct evidence of exposure, absorption, or pathway engagement in humans. In parallel, translational studies should establish clearer causal links to the fermentation process, bioactive formation, biological pathway activation, and clinical effects. A central task in this phase will be to distinguish more explicitly the contributions of the raw material, the fermentation process itself, microbial activity, matrix modification, and the reduction of antinutritional factors.

Medium-term research should also broaden the range of FF and populations under investigation. The PIMENTO reviews highlighted a clear asymmetry in the field: dairy-based fermented foods are comparatively well studied, evidence for some standardised plant-based fermented foodstuff is still emerging, whereas many traditional, artisanal, and region-specific FF remain poorly characterised and largely unexplored. This imbalance should be addressed. Future studies should include non-dairy and local FF, particularly those with potential nutritional relevance in settings shaped by plant-based dietary transitions or micronutrient deficiencies. This is especially relevant for FF that may contribute to nutrient adequacy in vulnerable populations or in groups shifting toward more plant-based diets. More attention should also be given to vulnerable or understudied groups, including children, older adults, and individuals with nutritional vulnerability, since these populations may be particularly informative for both benefit and risk assessment. When considering demographics, further considerations should also be given to inter-individual variability, including in microbiomes (48). To this end, future studies should integrate microbiome-stratified trials to identify responders and non-responders along with integrated, standardised frameworks to document host factors such as general diet, lifestyle, and exposome. Finally, in the medium term, holistic methods such as metagenomics, metabolomics, and proteomics should be well integrated to produce accepted endpoints similar to validated clinical endpoints.

#### 3.1.3 Long-term research roadmap: evidence consolidation and precision nutrition

In the longer term, the field should primarily move toward evidence consolidation with the ultimate objective being inclusion in precision nutrition strategies. Achieving this will require adequately powered, long-term randomised controlled trials using fully characterised FF interventions, appropriate comparators, dose–response evaluations, and follow-up investigations. Such studies are necessary to determine whether the modest and often short-term effects currently observed are sustained over time and whether they are clinically meaningful. A more ambitious long-term goal could be the development of a comprehensive, curated, open-access fermented foods database that integrates nutritional composition, relevant microbial strain-level information, fermentation derived metabolites, and variability across regions and production methods. Ideally, this database should eventually be integrated with dietary assessment systems and research groups/infrastructure relevant to FF. To gradually incorporate fermented foods into dietary recommendations, validations for biomarkers of FF intake and effect (both mechanistic and clinical ones) should be completed along with dietary assessment instruments beyond self-reporting/other current methods of measuring FF intake. Predictive AI-based models can then be employed to understand responses to fermented foods intake while integrating microbiome, genetic, metabolomic etc. data, and targeted FF-based dietary recommendations can be made. Longer-term observational strategies should also consider fermented foods not only as isolated products, but as part of whole dietary patterns, including total FF intake and, where possible, total dietary microbial exposure (102, 106). Finally, given the breadth of fermented foods and the possibilities they offer, combined with an ever-growing repertoire of holistic and AI-based analytical tools, topics like microbial interactions and synergistic effects in FF along with newer bioactives such as postbiotics and extracellular vesicles, should continue to be investigated.

### 3.2 Policy roadmap

#### 3.2.1 Short-term policy roadmap: foundations for standardisation and regulatory alignment

If FF are to progress from a heterogeneous research topic to a credible component of public health nutrition, policy development must advance alongside scientific standardisation. Currently, a major barrier is that many FF are poorly defined from a regulatory standpoint. Product categories vary across countries, classification systems are often imprecise, and studies do not always clearly distinguish between fermented and non-fermented products, foods containing live cultures and those in which microorganisms are no longer viable, or traditional and industrially standardised products. A primary policy priority is therefore the development of harmonised definitions and classification systems that enable FF to be identified more consistently in regulation, surveillance, and dietary guidance.

A second near-term priority is the establishment of clearer regulatory expectations for FF characterisation. Research and policy currently face the same challenge: a lack of minimum standards for describing the foods being evaluated. Policy frameworks should therefore support requirements for clearer reporting of the fermentation process, relevant strain-level microbial composition, viability at consumption where applicable, and the presence of hypothesised bioactive compounds or metabolites. This is also closely linked to labelling and transparency where improved labelling could contribute to both traceability and consumer understanding by clarifying whether a product is fermented, whether it contains live cultures, and what type of fermentation process was used. Public policy should be proactive in dispelling misinformation about fermented foods by promoting evidence-based communications, with frameworks in place for consumers and others to easily access curated FF-related information.

Policy development should also strengthen population level monitoring. National dietary surveys and intake tools often do not capture FF with sufficient precision, and broad categories such as fermented dairy remain too crude for meaningful exposure assessment with few exceptions (102). Fermented foods should therefore be integrated more explicitly into dietary surveillance systems and food classification tools. This would improve the quality of observational evidence, enable more reliable estimates of intake at the population level, and support future policy decisions about whether specific FF warrant targeted dietary guidance.

At the same time, safety frameworks should become more systematic. Fermented foods are often treated as historically safe, yet the evidence reviewed by PIMENTO WG3 indicates that safety should not simply be assumed (41). Policy should encourage more explicit adverse event monitoring in clinical studies and stronger surveillance of hazards associated with fermentation, including contamination and the accumulation of undesirable compounds. A more structured safety framework would strengthen public trust and align more closely with the expectation that efficacy, mechanism, and safety should be evaluated together rather than separately.

#### 3.2.2 Medium-term policy roadmap: evidence integration and regulatory pathways

In the medium-term, policy should support the development of enabling infrastructure. This includes global databases on FF composition, containing information on nutritional content, relevant microbial profiles, and fermentation-derived metabolites, as well as more harmonised frameworks for clinical trials. Importantly, such databases should be integrated into regulatory pipelines to ensure maximal standardisation. Guidance on comparator selection, product characterisation, dose, duration, and outcome assessment would improve comparability across studies and make future evidence more useful for translation into dietary guidance and regulatory purposes. In parallel, data sharing, open repositories, and collaboration among academia, regulators, and industry will be essential for the field to progress beyond isolated studies; endeavours fulfilling these criteria should be encouraged at the funding level. An important and often overlooked aspect of research, particularly health-oriented research, is health equity. As mentioned above, fermented food-based research (particularly underrepresented FF) in underrepresented demographics and regions, as well as vulnerable populations, should be supported by appropriate policies. Ideally, such policies and research can contribute to dietary guidelines that are not only scientifically sound but also culturally relevant.

#### 3.2.3 Long-term policy roadmap: integration into public health and precision nutrition

Over the longer term, and contingent on substantially strengthened evidence, these steps could support more evidence-based dietary recommendations for fermented foods and clearer regulatory pathways for health claims. Regarding the former, establishing a recommended daily intake of microbes (with or without fermented foods as a vehicle) and integrating FF into national dietary guidelines, further harmonised into international guidelines, is a major objective. Regulatory standardisation in the short- and medium-term should lead to streamlined approval processes for functional fermented foods. Relatedly, more policy level support should be provided for precision or targeted FF with defined starter cultures or microbial consortia, tailored to specific populations with health conditions. Indeed, in time, policy should encourage the integration of fermented foods into preventive healthcare strategies in the public sphere, in combination with microbiome-based/informed health strategies, including in suboptimal health prevention frameworks for cardiovascular and gut health, metabolic syndrome, and related conditions. Ultimately, these efforts align with precision nutrition goals, which have been a long-term objective in public health, and policy should reflect research that would enable integration of FF into targeted public nutrition strategies. Importantly, such policy developments should be based only on sufficiently characterised products, taking advantage of fermented foods microbiomics/metabolomics data, and supported by more coherent human evidence.

## 4 Limitations

The Roadmap’s conclusions, while rigorous, should be read in light of several considerations regarding its evidence base. For example, the coverage is uneven across fermented-food categories, populations, and regions, with dairy-based products and adult populations in high-income settings comparatively over-represented relative to other foods, life stages, and geographies. Alcoholic fermented beverages were also excluded from most component reviews, further narrowing the generalisability of the Roadmap’s conclusions to non-alcoholic fermented foods. Additionally, the regulatory and evidentiary framework applied throughout, i.e., EFSA’s three-tier evidence-certainty system and EU health-claim regulation, is European in orientation and may not transfer directly to other jurisdictions. The 17 underlying PIMENTO WG3 reviews also differ in design (systematic, scoping, narrative), search strategy, and risk-of-bias tool (Supplementary Table S1); assessment of publication bias and selective outcome reporting was similarly not applied uniformly across the 17 reviews, so review-level findings were synthesised qualitatively rather than pooled quantitatively. This evidence base is, moreover, drawn specifically from the 17 PIMENTO WG3 reviews, each conducted under a registered protocol and harmonised systematic methodology, rather than from an independently commissioned umbrella review of the wider literature; while this design ensured methodological consistency and drew on the work of 141 co-authors across multiple institutions. It further means that the conclusions of the Roadmap are bounded by the scope of the PIMENTO WG3 programme rather than benchmarked against an external, independently assembled evidence base.

## 5 Conclusion

The activities of PIMENTO WG3 represent a coordinated, multiyear effort rather than a conventional narrative roadmap. Through one position paper and 17 review-based outputs, the initiative enabled synthesis across human evidence, mechanisms of action, bioavailability, food characterisation, and safety. This scale allowed recurring methodological and translational limitations to be identified across the fermented food field as a whole, rather than within isolated review topics alone. While this work suggests potential health benefits, the overall strength of the evidence remains limited due to its heterogeneous and often incomplete nature. Advancing the field will require more rigorous and harmonised research methodologies with future studies addressing clearly defined research questions, supported by mechanistically informed *in vitro* and *in vivo* experimental designs. Efforts should be potentially strategically focused on a narrower set of research programmes targeting fermented foods, or specific FF components or groups, with the most promising functional properties (Figure 2). These programmes should integrate clinical outcomes, underlying mechanisms of action, and comprehensive product characterisation including safety, composition, and stability of bioactive compounds. Greater process control and standardisation are needed to generate reproducible evidence on the health effects of fermented foods, but this should not imply narrowing the field to a limited set of industrially simplified products. The diversity of traditional, regional, and artisanal FF and their fermentation processes should also be maintained, both as culturally relevant foods and as reservoirs of microbial and metabolic diversity for future innovation. Formal evaluation and approval of fermented-food-specific health claims by regulatory bodies such as EFSA and the FDA, and the inclusion of fermented-food-specific guidance in national or international dietary policy, can potentially act as proxy measurable outcomes for progress on the proposed roadmap. Overall, at the research level, the proposed roadmap may enable a transition from fragmented, poorly characterised, association-based evidence towards standardised, mechanistically validated, and regulatory-grade science regarding fermented foods and their effects on health, while the policy roadmap elevates FF in a social context, from historically safe but poorly defined foods to well-characterised, regulated, and evidence-based components of public health nutrition. To our knowledge, this roadmap represents the first proposition of its kind for the fermented foods field: a comprehensive, evidence-based synthesis of findings and knowledge gaps across the full range of health domains examined by PIMENTO WG3, translated into a timely, unified, foundational and strategic framework for future research and policy action.

## Group members of the PIMENTO health benefits and risks working group

The following individuals served as collaborators and site investigators for the PIMENTO Health Benefits and Risks Working Group. They participated in the definition of the content of the manuscript and reviewed and revised the manuscript. Signe Adamberg, Department of Chemistry and Biotechnology, Tallinn University of Technology, Tallinn, Estonia; Elizabeth Adesemoye, Department of Microbiology, Faculty of Life Science, Federal University Oye Ekiti, Ekiti State, Nigeria; Fojan Agahi, Ciències de l’Alimentació, Toxicologia i Medicina Legal, Departament de Medicina Preventiva i Salut Pública, Universidad de Valencia, Valencia, Spain; Zeynep Agirbasli, Department of Food Engineering, Izmir Institute of Technology, Izmir, Türkiye; Ecem Akan, Department of Dairy Technology, Faculty of Agriculture, Aydın Adnan Menderes University, Aydın, Türkiye; Asli Akpinar, Department of Food Engineering, Faculty of Engineering and Natural Science, Manisa Celal Bayar University, Manisa, Türkiye; Panagiota Alvanoudi, Laboratory of Food Chemistry and Technology, School of Chemistry, Aristotle University of Thessaloniki, Thessaloniki, Greece; Emilia Alves, CBIOS Research Center for Biosciences & Health Technologies, Universidade Lusófona, Lisbon, Portugal; Ljupco Angelovski, Faculty of Veterinary Medicine Skopje, Saints Cyril and Methodius University of Skopje, Skopje, North Macedonia; Elena Arranz, Institute of Food Science Research (CIAL, CSIC-UAM), Universidad Autónoma de Madrid (CEI UAM + CSIC), Madrid, Spain; Sultan Arslan-Tontul, Food Engineering Department, Agricultural Faculty, Selcuk University, Konya, Türkiye; Ricardo Assunção, Food and Nutrition Department, National Institute of Health Dr. Ricardo Jorge, Lisbon, Portugal, Egas Moniz Center for Interdisciplinary Research (CiiEM), Egas Moniz School of Health and Science, Almada, Portugal; Erfan Bagherzadehsurbagh, Department of Food Engineering, Institute of Natural and Applied Sciences, Akdeniz University, Antalya, Türkiye; Nurcan Bağlam, Nutrition and Dietetics Department, Faculty of Health Science, Sivas Cumhuriyet University, Sivas, Türkiye; Cornelia Bär, Competence Division Methods Development and Analytics, Biochemistry of Milk and Microorganisms, Agroscope, Bern, Switzerland; Simona Lucia Bavaro, Istituto di Scienze delle Produzioni Alimentari, Consiglio Nazionale delle Ricerche, Grugliasco (To), Italy; Miona Belović, Institute of Food Technology, University of Novi Sad, Novi Sad, Serbia; Muzeyyen Berkel Kasikci, Department of Food Engineering, Faculty of Engineering and Natural Science, Manisa Celal Bayar University, Manisa, Türkiye; Thomas Bintsis, Faculty of Veterinary Medicine, Aristotle University of Thessaloniki, Thessaloniki, Greece; Grégory Bouchaud, Biopolymères Intéractions Assemblages (BIA), INRAE, Nantes, France; Tugce Bulmus-Tuccar, Yuksek Ihtisas Universitesi, Ankara, Türkiye; Kathryn J. Burton-Pimentel, Food Microbial Systems Research Division, Agroscope, Bern, Switzerland; Özge Cemali, Department of Nutrition and Dietetics, Faculty of Health Sciences, Trakya University, Edirne, Türkiye; Marie-Christine Champomier-Vergès, Université Paris-Saclay, INRAE, AgroParisTech, Micalis Institute, Jouy-en-Josas, France; Emilie Chanséaume-Bussiere, Nutrifizz, Clermont-Ferrand, France; Christophe Chassard, Unité Mixte de recherche sur le Fromage, INRAE, UCA, VetAgro Sup, Aurillac, France; Zuzana Ciesarová, Food Research Institute, National Agricultural and Food Centre, Bratislava, Slovakia; Paul Cotter, APC Microbiome Ireland, Cork, Ireland, Department of Food Biosciences, Teagasc Food Research Centre, Fermoy, Co. Cork, Ireland, VistaMilk, Cork, Ireland; Hülya Cunedioglu, Department of Agriculture, Food and Natural Science Engineering, University of Foggia, Termoli, Italy; Biljana Cvetković, Institute of Food Technology, University of Novi Sad, Novi Sad, Serbia; Bojana Danilovic, University of Nis, Faculty of Technology, Leskovac, Serbia; Muriel Derrien, VIB-Center of Microbiology, KU Leuven, Leuven, Belgium; Ioannis Diamantoglou, Charokopeio Panepistemio, Athens, Greece; Emine Dincer, Nutrition and Dietetics Department, Faculty of Health Science, Sivas Cumhuriyet University, Sivas, Türkiye; Julieta Domínguez-Soberanes, Facultad de Ingeniería, Universidad Panamericana, Aguascalientes, Mexico; Gamze Düven, Milk and Dairy Technology Programme, Department of Food Processing, Karacabey Vocational School, Bursa Uludag Universitesi, Bursa, Türkiye; Mounaim Halim El Jalil, Higher School of Technology, Mohammed V University of Rabat, Rabat, Morocco; Erenay Erem, Department of Food Engineering, Faculty of Chemical Metallurgical Engineering, Istanbul Technical University, Istanbul, Türkiye; Tuba Esatbeyoglu, Department of Molecular Food Chemistry and Food Development, Institute of Food and One Health, Gottfried Wilhelm Leibniz University Hannover, Hannover, Germany; Anthony Fardet, INRAE, UMR 1019, Unité de Nutrition Humaine, Université Clermont Auvergne, Clermont-Ferrand, France; Dominic N. Farsi, Department of Nutritional Sciences, King’s College London, London, United Kingdom; Pavli Foteini, Department of Food Sciences and Nutrition, University of Malta, Msida, Malta; Juana Frias, Institute of Food Science, Technology and Nutrition, Spanish National Research Council (ICTAN-CSIC), Madrid, Spain; Mario Caruana Grech Perry, Department of Food Sciences, Nutrition & Dietetics, Faculty of Health Sciences, University of Malta, Malta; Amparo Gamero, Bionutest research group, Faculty of Pharmacy and Food Sciences, Dep. Preventive Medicine and Public Health, Food Science, Toxicology and Forensic Medicine, University of Valencia, Burjassot, Valencia, Spain; Mónica Gandía, Bionutest research group, Faculty of Pharmacy and Food Sciences, Dep. Preventive Medicine and Public Health, Food Science, Toxicology and Forensic Medicine, University of Valencia, Burjassot, Valencia, Spain; Enriqueta Garcia-Gutierrez, Agronomic Engineering Department, Technical University of Cartagena, Cartagena, Spain; Amaury Gérard, Brewing & Food Industry Research Unit, LABIRIS, Anderlecht, Belgium; Aiswarya Girija, Institute of Biological, Environmental and Rural Sciences (IBERS), Aberystwyth University, Wales, United Kingdom; Carmen M. Gonzalez-Domenech, Department of Microbiology, School of Medicine, University of Malaga, Málaga, Spain; Ziba Güley, Department of Food Engineering, Faculty of Engineering, Alanya Alaaddin Keykubat University, Alanya, Antalya, Türkiye; Zehra Gulsunoglu-Konuskan, Nutrition and Dietetics Department, Faculty of Health Sciences, Istanbul Aydin University, Istanbul, Türkiye; Burcu Gündüz Ergün, Department of Molecular Biology and Genetics, Faculty of Arts and Sciences, Yildiz Technical University, Istanbul, Türkiye, Health Biotechnology Centre of Excellence for Joint Research and Application (SABIOTEK), Yildiz Technical University, Istanbul, Türkiye; Carolina Gutiérrez-Repiso, Centro de Investigación Biomédica en Red de la Fisiopatología de la Obesidad y Nutrición (CIBEROBN), Instituto de Salud Carlos III, Madrid, Spain, Department of Endocrinology and Nutrition Virgen de la Victoria University Hospital, Málaga, Spain, Instituto de Investigación Biomédica de Málaga y Plataforma en Nanomedicina-IBIMA Plataforma BIONAND, Málaga, Spain; Mustafa Guzel, Department of Food Engineering, Hitit University, Corum, Türkiye; Hayriye S. Harsa, Department Food Engineering, Faculty of Engineering, Izmir Institute of Technology, Urla, İzmir, Türkiye; Daniel Heine, School of Agricultural, Forest and Food Sciences HAFL, Bern University of Applied Sciences, Zollikofen, Switzerland; Elaine Hillesheim, Food Microbial Systems Research Division, Agroscope, Bern, Switzerland; Melanie Huch, Institute of Nutritional and Food Microbiology, Max Rubner-Institut, Karlsruhe, Germany; Christèle Humblot, QualiSud, Université de Montpellier, Avignon Université, CIRAD, Institut Agro, IRD, Université de la Réunion, Montpellier, France, French National Research Institute for Sustainable Development (IRD), Montpellier, France; Bahtir Hyseni, Faculty of Food Technology, University “Isa Boletini,” Mitrovica, Republic of Kosovo; Aline Issa, Faculty of Nursing and Health Sciences, Notre Dame University-Louaize, Zouk Mosbeh, Lebanon; Sumeye Kabakci, National Food Reference Laboratory, Ministry of Agriculture and Forestry, Ankara, Türkiye; Minna Kahala, Natural Resources Institute Finland (Luke), Helsinki, Finland; Hatice Kalkan Yıldırım, Department of Food Engineering, Faculty of Engineering, Ege University, Izmir, Türkiye; Aggeliki Kalogeropoulou, Laboratory of Food Chemistry and Technology, School of Chemistry, Aristotle University of Thessaloniki, Thessaloniki, Greece; Mustafa F. Karagöz, Hitit Universitesi, Çorum, Türkiye; Barçın Karakaş-Budak, Department of Food Engineering, Faculty of Engineering, Akdeniz University, Antalya, Türkiye; Sibel Karakaya, Department of Food Engineering, Faculty of Engineering, Ege University, Izmir, Türkiye; Harun Kesenkas, Department of Dairy Technology, Faculty of Agriculture, Ege University, Izmir, Türkiye; Erhan Keyvan, Department of Food Hygiene and Technology, Faculty of Veterinary Medicine, Burdur Mehmet Akif Ersoy University, Burdur, Türkiye; Meral Kilic-Akyilmaz, Department of Food Engineering, Faculty of Chemical Metallurgical Engineering, Istanbul Technical University, Istanbul, Türkiye; Gabriela Krausova, Dairy Research Institute, Prague, Czechia; Ibrahim E. Künili, Department of Fishing and Fish Processing Technology, Faculty of Marine Sciences and Technology, Canakkale Onsekiz Mart University, Canakkale, Türkiye; Mary-Liis Kütt, äio tech OÜ, Tallinn, Estonia; Marta Laranjo, MED-Mediterranean Institute for Agriculture, Environment and Development & CHANGE-Global Change and Sustainability Institute, Departamento de Medicina Veterinária, Escola de Ciências e Tecnologia, Universidade de Évora, Évora, Portugal; Sandrine Louis, Institute of Nutritional and Food Microbiology, Max Rubner-Institut, Karlsruhe, Germany; Jeadran Malagon-Rojas, Maestría en Salud Pública, El Bosque University, Bogotá, Colombia, Instituto Nacional de Salud de Colombia, Bogotá, Colombia; Fani Th. Mantzouridou, Laboratory of Food Chemistry and Technology, School of Chemistry, Aristotle University of Thessaloniki, Thessaloniki, Greece; Julie Mardon, Université Clermont Auvergne, INRAE, VetAgro Sup, UMR 0545 Fromage, Lempdes, France; Lidia Markiewicz, Immunology and Food Microbiology Group, Institute of Animal Reproduction and Food Research, Polish Academy of Sciences, Olsztyn, Poland; Cristina Martínez-Villaluenga, Institute of Food Science, Technology and Nutrition, Spanish National Research Council (ICTAN-CSIC), Madrid, Spain; Antonia Matalas, Department of Nutrition and Dietetics, School of Health Sciences and Education, Harokopio University, Athens, Greece; Baltasar Mayo, Departamento de Microbiología y Bioquímica, Instituto de Productos Lácteos de Asturias (IPLA), Consejo Superior de Investigaciones Científicas (CSIC), Oviedo, Spain; Ryma Merabti, Laboratory of Biotechnology and Food Quality (Bioqual), Institute of Nutrition, Food and Agri-Food Technologies (INATAA), University Constantine 1, Algeria; Victoria Meslier, Université Paris-Saclay, INRAE, Metagenopolis, Jouy-en-Josas, France; Sandra Mojsova, Faculty of Veterinary Medicine Skopje, Saints Cyril and Methodius University of Skopje, Skopje, North Macedonia; Isabel Moreno-Indias, CIBER in Physiopathology of Obesity and Nutrition (CIBEROBN), Carlos III Health Institute, Madrid, Spain; Arghya Mukherjee, APC Microbiome Ireland, Cork, Ireland, Department of Food Biosciences, Teagasc Food Research Centre, Fermoy, Co. Cork, Ireland; Eleni Naziri, Department of Food Science and Nutrition, School of Environment, University of the Aegean, Lemnos, Greece; Anastasios Nikolaou, Department of Molecular Biology and Genetics, Democritus University of Thrace, Alexandroupolis, Greece; Daniela Nikolovska Nedelkoska, Faculty of Technology and Technical Sciences, University St. Kliment Ohridski - Bitola, Veles, North Macedonia; Anastasia Ntantou, Department of Nutrition and Dietetics, School of Health Sciences and Education, Harokopio University, Athens, Greece; Sine Özmen Toğay, Department of Food Engineering, Faculty of Agriculture, Bursa Uludag University, Bursa, Türkiye; Sebnem Ozturkoglu-Budak, Department of Dairy Technology, Faculty of Agriculture, Ankara University, Ankara, Türkiye; Vasfiye H. Özyurt, Department of Gastronomy and Culinary Arts, Mugla Sitki Kocman University, Mugla, Türkiye; Zada Pajalic, Department of Nursing and Health Promotion, Faculty of Health Sciences, Oslo Metropolitan University, Oslo, Norway; Recep Palamutoglu, Department of Nutrition and Dietetics, Faculty of Health Sciences, Afyonkarahisar Health Sciences University, Afyonkarahisar, Türkiye; Demosthenes B. Panagiotakos, Department of Nutrition and Dietetics, School of Health Sciences and Education, Harokopio University, Athens, Greece; Konstantinos Papadimitriou, Department of Food Science and Human Nutrition, Agricultural University of Athens, Athens, Greece; Alejandra Parada-López, Grupo de Salud Ambiental y Laboral, Instituto Nacional de Salud, Bogotá, Colombia; Diana Paveljšek, University of Ljubljana, Biotechnical Faculty, Ljubljana, Slovenia; Elena Peñas, Institute of Food Science, Technology and Nutrition, Spanish National Research Council (ICTAN-CSIC), Madrid, Spain; Vincenzo Pennone, Cell and Tissue Engineering Laboratory, IRCCS Istituto Ortopedico Galeazzi, Milan, Italy; Eugenia Pertziger, (1) Department of Epidemiology and Health Systems, Center for Primary Care and Public Health (Unisanté), University of Lausanne, Lausanne, Switzerland, (2) Food Microbial Systems Research Division, Agroscope, Bern, Switzerland; Smilja Praćer, Department of Neurobiology, Institute for Biological Research “Sinisa Stankovic,” National Institute of the Republic of Serbia, University of Belgrade, Belgrade, Serbia; Reimo Priidik, Wizon OÜ, Tallinn, Estonia; Milica Prvulovic, Department of Neurobiology, Institute for Biological Research “Sinisa Stankovic,” National Institute of the Republic of Serbia, University of Belgrade, Belgrade, Serbia; Endra Luzha-Pula, Food Hygiene and Technology Department, Faculty of Veterinary Medicine, Istanbul University-Cerrahpasa, İstanbul, Türkiye; Catarina Rosado, CBIOS Research Center for Biosciences & Health Technologies, Universidade Lusófona, Lisbon, Portugal; Patricia Ruiz-Limón, CIBER in Physiopathology of Obesity and Nutrition (CIBEROBN), Carlos III Health Institute, Madrid, Spain; Pasquale Russo, Department of Food, Environmental and Nutritional Sciences, University of Milan, Milan, Italy; Helen Saar, Research Services, AS TFTAK, Tallinn, Estonia; José Ángel Salas-Millán, APC Microbiome Ireland, Cork, Ireland; Seppo Salminen, Food and Nutrition Research Center, Faculty of Medicine, University of Turku, Turku, Finland; Elisa Salvetti, Dipartimento di Biotecnologie, Universita degli Studi di Verona, Verona, Italy; Dushica Santa, Faculty of Agricultural Sciences and Food, Saints Cyril and Methodius University of Skopje, Skopje, North Macedonia; Taner Sar, Swedish Centre for Resource Recovery, University of Borås, Borås, Sweden; Inga Sarand, Department of Chemistry and Biotechnology, Tallinn University of Technology, Tallinn, Estonia; Isabelle Savary-Auzeloux, INRAE, UMR 1019, Unité de Nutrition Humaine, Université Clermont Auvergne, Clermont-Ferrand, France; Else Schneider, Center of Affective, Stress and Sleep Disorders (ZASS), University Psychiatric Clinics (UPK), University of Basel, Basel, Switzerland; Beatriz N. Silva, Universidade Católica Portuguesa, CBQF - Centro de Biotecnologia e Química Fina – Laboratório Associado, Escola Superior de Biotecnologia, Porto, Portugal; Valentina Simeunović, Department of Neurobiology, Institute for Biological Research “Sinisa Stankovic,” National Institute of the Republic of Serbia, University of Belgrade, Belgrade, Serbia; Laurencia Songré-Ouattara, Centre National de la Recherche Scientifique et Technologique, Ouagadougou, Burkina Faso; Małgorzata Starowicz, Chemistry and Biodynamics of Food Team, Institute of Animal Reproduction and Food Research of the Polish Academy of Sciences, Olsztyn, Poland; Dominic Stoll, Institute of Nutritional and Food Microbiology, Max Rubner-Institut, Karlsruhe, Germany; Michail Syrpas, Department of Food Science and Technology, Kaunas University of Technology, Kaunas, Lithuania; Hania Szajewska, Department of Paediatrics, Medical University of Warsaw, Warsaw, Poland; Julien Tap, Université Paris-Saclay, INRAE, AgroParisTech, Micalis Institute, Jouy-en-Josas, France; Ayşe Nur Tonay, UCD School of Biosystems and Food Engineering, University College Dublin, Dublin, Ireland; Thomy Tonia, Institute of Social and Preventive Medicine, University of Bern, Bern, Switzerland; Aleksandra Torbica, Institute of Food Technology, University of Novi Sad, Novi Sad, Serbia; Biljana Trajkovska, Faculty of Biotechnical Sciences, University st. Kliment Ohridski, Bitola, North Macedonia; Guy Vergères, Food Microbial Systems Research Division, Agroscope, Bern, Switzerland; Bojana Vidovic, Department of Bromatology, Faculty of Pharmacy, University of Belgrade, Belgrade, Serbia; Andjela Vukojevic, Institute for Biological Research “Sinisa Stankovic,” National Institute of Republic of Serbia, University of Belgrade, Belgrade, Serbia; Oktay Yerlikaya, Department of Dairy Technology, Faculty of Agriculture, Ege University, Izmir, Türkiye; Aycan Yigit Cinar, Department of Food Engineering, Faculty of Engineering and Natural Sciences, Bursa Technical University, Bursa, Türkiye; Birsen Yilmaz, Advanced Research Unit on Metabolism, Development and Ageing (ARUMDA), Tata Institute of Fundamental Research, Hyderabad, India, Tata Institute of Fundamental Research Hyderabad, Hyderabad, India; Arkadiusz Józef Zakrzewski, Department of Food Microbiology, Meat Technology and Chemistry, University of Warmia and Mazury in Olsztyn, Olsztyn, Poland; Zsolt Zalán, Institute of Food Science and Technology, Hungarian University of Agriculture and Life Sciences, Budapest, Hungary.
