# Supplementary material for: Health benefits of fermented foods: a strategic roadmap from the PIMENTO initiative
Source: Front Nutr. 2026 Sep 18;13:1904521. doi: 10.3389/fnut.2026.1904521 (PMC13630987; doi:10.3389/fnut.2026.1904521)
Supplement: Supplementary file 1 [file Data_Sheet_1.pdf]

Supplementary Data

Health Benefits of Fermented Foods: A Strategic Roadmap from the PIMENTO Initiative

Mustafa Guzel[1,*,#], Arghya Mukherjee[2,3,#,*], Ricardo Assunção[4], Juana Frias[5], Christèle Humblot[6,7], Marta Laranjo[8], Isabelle Savary-Auzeloux[9], Birsen Yilmaz[10,11], Christophe Chassard[12], PIMENTO Health Benefits & Risks Working Group[§], Smilja Praćer[13,‡], Guy Vergères[14, ‡]

[1]Department of Food Engineering, Hitit University, Corum, Türkiye, [2]APC Microbiome Ireland, Cork , Ireland, [3]Department of Food Biosciences, Teagasc Food Research Centre, Fermoy, Co. Cork, Ireland, [4]Food and Nutrition Department, National Institute of Health Dr. Ricardo Jorge, Lisbon, Portugal, [5]Institute of Food Science, Technology and Nutrition, Spanish National Research Council (ICTAN-CSIC), Madrid, Spain, [6]QualiSud, Université de Montpellier, Avignon Université, CIRAD, Institut Agro, IRD, Université de la Réunion, Montpellier, France, [7]French National Research Institute for Sustainable Development (IRD), Montpellier, France, [8]MED-Mediterranean Institute for Agriculture, Environment and Development & CHANGE-Global Change and Sustainability Institute, Departamento de Medicina Veterinária, Escola de Ciências e Tecnologia, Universidade de Évora, Évora, Portugal, [9]INRAE, UMR 1019, UNH, CRNH Auvergne, Clermont-Ferrand, France, [10]Advanced Research Unit on Metabolism, Development and Ageing (ARUMDA), Tata Institute of Fundamental Research, Hyderabad, India, [11]Tata Institute of Fundamental Research Hyderabad, Hyderabad, India, [12]Unité Mixte de recherche sur le Fromage, INRAE, UCA, VetAgro Sup, Aurillac, France, [13]Department of Neurobiology, Institute for Biological Research "Sinisa Stankovic", National Institute of the Republic of Serbia, University of Belgrade, Belgrade, Serbia, [14]Food Microbial Systems Research Division, Agroscope, Bern, Switzerland.

[#]These authors contributed equally as first co-authors.
[‡]Last co-authors.
[§]Members of the PIMENTO Health Benefits & Risks Working Group are provided at the end of the main manuscript.
*Correspondence: Mustafa Guzel, musguzel@gmail.com; Arghya Mukherjee, arghya.mukherjee@teagasc.ie

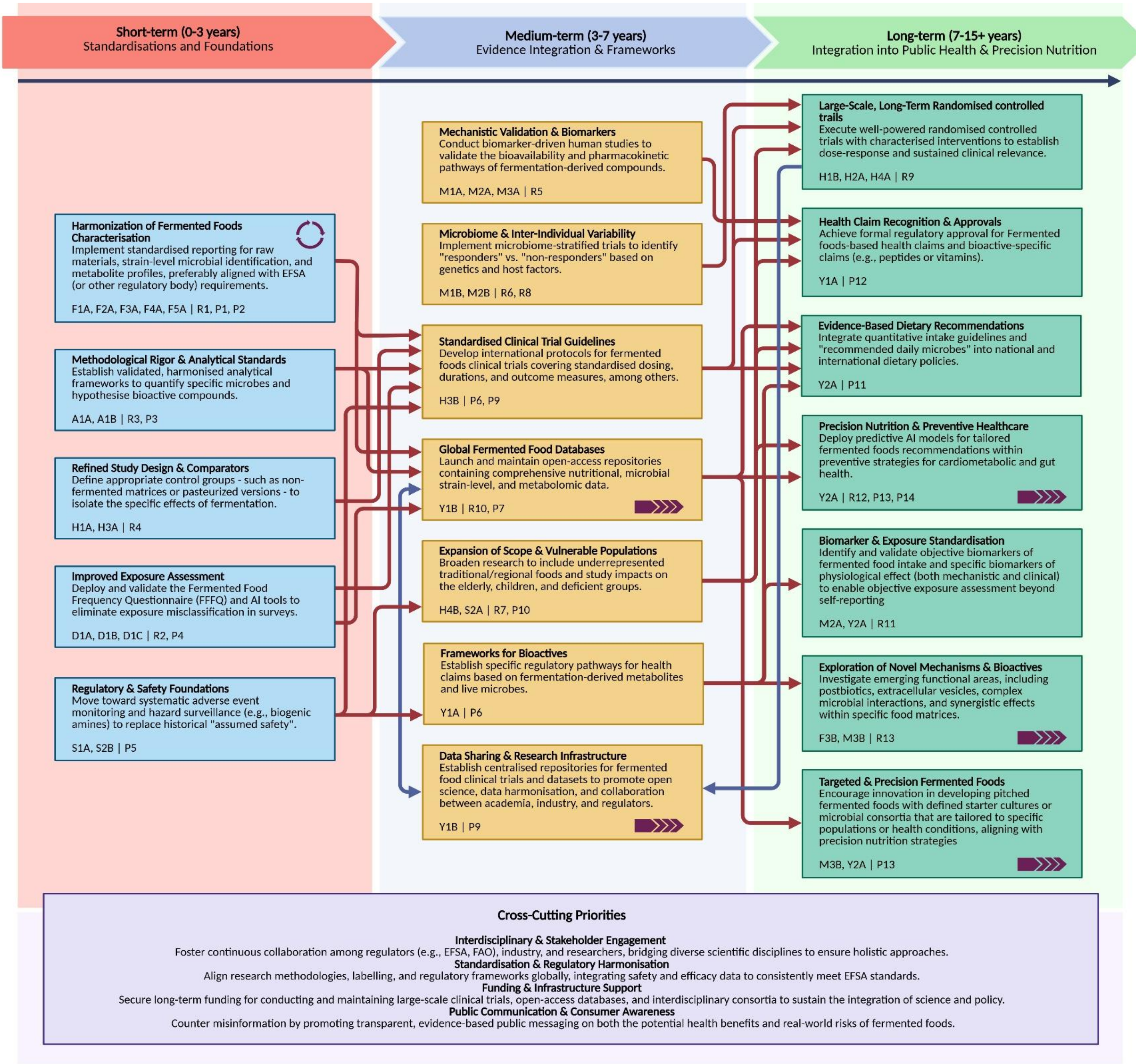

Supplementary Figure S1. Detailed strategic research and policy roadmap for fermented foods. This figure presents the full underlying detail of the strategic roadmap summarised in Figure 1, illustrating the transition required to move from fragmented, poorly characterised, association-based evidence in fermented foods research toward standardised, mechanistically validated, regulatory-grade science and evidence-based public health nutrition. Timeframes for roadmap objectives are mapped on the horizontal axis and structured across three chronological phases, colour-coded by chevron: short-term (0–3 years, blue), focused on standardisation and foundational tools; medium-term (3–7 years, gold), targeting mechanistic validation and evidence integration; and long-term (7–15+ years, mint), aiming at large-scale evidence consolidation and precision nutrition. Discrete boxes represent deliverables, with connecting arrows (maroon) mapping critical dependencies and illustrating how foundational actions are prerequisites for building global databases, conducting large-scale clinical trials, and eventually achieving health claim approvals and evidence-based dietary recommendations. Actions that can act retroactively or are mutually dependent are indicated by blue arrows. The bracketed tags within each action box denote the specific EFSA-guided domain framework items fulfilled by that action, and indicate exactly which Research Roadmap (R1-R13) and Policy Roadmap (P1-P14) objectives are addressed; some

actions fulfil both research and policy objectives and are labelled accordingly. The major EFSA-guided Domains are: Food Characterisation (F), Analytical Methods (A), Dietary Assessment (D), Human Evidence (H), Mechanisms (M), Safety (S), and Synthesis & Integration (Y), with Subdomains comprising Raw Materials & Composition (F1), Food Diversity & Cultural Scope (F2), Manufacturing Process (F3), End-Product Profiling (F4), Stability & Delivered Exposure (F5), Analytical Methodologies (A1), Exposure Tracking (D1), Specificity & Magnitude of Effect (H1), Dose-Response (H2), Study Design & Controls (H3), Population & Sustainability (H4), Bioavailability (M1), Causal Linkages (M2), Vitamins (M3), Clinical Tolerability (S1), Vulnerability & Risk (S2), Regulatory Submissions (Y1), and Precision Nutrition (Y2); further detail is provided in Supplementary Table S2. Cross-cutting priorities that underpin the roadmap across all timeframes are shown as a separate domain and include continuous interdisciplinary stakeholder engagement, transparent public communication, and sustained infrastructure funding, among others. Actions expected to continue beyond their designated timeframe are indicated by purple progress chevrons at the base of the corresponding box, and actions anticipated to be iteratively refined are indicated by a purple circular progress icon. This detailed roadmap corresponds directly to the simplified summary presented in Figure 1, where the measurable short-, medium-, and long-term milestones associated with each phase are described in full.

Supplementary Table S1. **Detailed summary of PIMENTO WG3 reviews.** The details of study scope, principal findings, evidence statements, and EFSA-relevant interpretations as evidenced in the PIMENTO WG3 reviews is outlined in a tabular representation below. For additional information please refer to Table 1 in the main text as well as the original publications.

<table>
<tr><th colspan="9">E1. Mukherjee et al. (2025). Impact of fermented foods consumption on gastrointestinal wellbeing in healthy adults: a systematic review and meta-analysis (1).</th></tr>
<tr><th>Number of papers</th><th>Meta-analysis</th><th>Type of FF most studied</th><th>Population</th><th>Main outcome</th><th>Specific outcomes</th><th>Main Results</th><th>Evidence</th><th>Comments / EFSA allegation</th></tr>
<tr><td>25 papers: 4 non-randomized studies, 1 observational study, 2 randomized crossover studies (RCoT), 19 RCTs</td><td>19 RCTs used for meta-analysis</td><td>Fermented foods as a whole, with strong representation of fermented dairy products; plant-derived fermented foods were also included</td><td>Healthy adults</td><td>Gastrointestinal (bowel function)</td><td>Bowel frequency; stool consistency; stool pH; stool water content; stool bulk; intestinal transit time; abdominal pain; bloating; borborygmi; flatulence; constipation incidence and severity; incomplete evacuation; straining during defecation</td><td>Fermented food consumption may beneficially affect several bowel-function and gastrointestinal-wellbeing outcomes, including stool consistency, hard stools, transit time, abdominal symptoms, and constipation-related measures. However, certainty varied substantially across endpoints.</td><td>Certainty of evidence was highly variable and mostly low.</td><td>The evidence base remains limited by underrepresentation of traditional and non-dairy fermented foods and by the lack of vertically integrated mechanistic studies; overall, the evidence is neither convincing nor sufficient for an EFSA-style claim.</td></tr>
<tr><th>Review type</th><th>Search date range</th><th colspan="2">Risk-of-bias tool</th><th colspan="2">Comparator</th><th colspan="3">Limitations</th></tr>
<tr><td>Systematic review + meta-analysis</td><td>To 31 Jan 2025</td><td colspan="2">Not confirmed for individual studies (GRADE used for certainty)</td><td colspan="2">Non-fermented equivalent (Table 1 PICO)</td><td colspan="3">Yes — Section 8: excludes alcoholic FF and meat-based FF, English-only, one database excluded</td></tr>
</table>

E2. Hyseni *et al.* (2026). The role of fermented foods in managing food allergies in children and adults: a systematic review (2).								
**Number of papers**	**Meta-analysis**	**Type of FF most studied**	**Population**	**Main outcome**	**Specific outcomes**	**Main Results**	**Evidence**	**Comments / EFSA allegation**
10 studies (7 PICO, 3 PIO)	No	Fermented food products relevant to food allergy, including soy products, gluten-free sourdough wheat baked goods, fruit products, novel plant-based products, and milk products	Children and adults (6 months-65 years 172 old) with food allergies, including IgE-mediated, non-IgE-mediated, or mixed mechanisms	Food allergies	Allergic symptoms, growth parameters, and the prevalence of food allergies following fermented food consumption.	Fermentation often reduced allergenicity, particularly in gluten- and soy-based products, and some studies suggested immunomodulatory effects and improved tolerance. However, results were heterogeneous, and one study reported worsened symptoms with fermented soy sauce.	Evidence suggests potential benefit but remains limited and methodologically heterogeneous	Fermented foods may have a role in reducing allergenicity and supporting food allergy management, but current evidence is insufficient for EFSA-style substantiation and requires better human trials with mechanistic confirmation.
**Review type**	**Search date range**	**Risk-of-bias tool**		**Comparator**		**Limitations**		
Systematic review	1 Jan 1970 – 31 Dec 2024	Not reported		Not explicitly defined; lack of comparator noted as a limitation in some included studies		Evidence base is small-scale, underpowered, and methodologically limited, precluding firm conclusions for regulatory consideration		

E3. Akpinar *et al.* (2025). Efficacy of fermented foods for the prevention and treatment of bacterial vaginosis and vulvovaginal candidiasis (3).								
**Number of papers**	**Meta-analysis**	**Type of FF most studied**	**Population**	**Main outcome**	**Specific outcomes**	**Main Results**	**Evidence**	**Comments / EFSA allegation**
9 (6 interventional (2 cross over, 3 RCT, 1 non RCT) 3 observational)	No	Yogurt, fermented milk	Women with or without bacterial vaginosis and/or vulvovaginal candidiasis; some studies included women with HIV	Prevention and treatment of bacterial vaginosis and vulvovaginal candidiasis	Bacterial vaginosis; vulvovaginal candidiasis	Human studies generally reported positive effects, but the evidence base was limited and focused almost exclusively on fermented dairy products	Evidence is limited and controls were often inadequate.	Human studies remain limited and largely restricted to fermented dairy products; despite promising findings, current evidence is neither convincing nor sufficient to establish a causal relationship.
**Review type**	**Search date range**	**Risk-of-bias tool**		**Comparator**		**Limitations**		
Systematic review (2-phase)	To 31 Aug 2023, updated to 31 Dec 2024	Cochrane RoB2 (RCTs) + modified Newcastle-Ottawa Scale (observational)		Defined PICO comparator (detailed in supplementary)		Included trials had limited participant numbers, brief intervention durations, and pilot/short-report designs; recent fermented-food-specific trials are scarce		

E4. Starowicz *et al.* (2026). Effects of sourdough and yeast fermented breads consumption on glycaemia in healthy populations: a systematic review (4).								
**Number of papers**	**Meta-analysis**	**Type of FF most studied**	**Population**	**Main outcome**	**Specific outcomes**	**Main Results**	**Evidence**	**Comments / EFSA allegation**
27 RCTs	No	Fermented bread	Adults aged 18 years and older, predominantly healthy; some studies included participants with high BMI, prediabetes, or impaired glucose tolerance	Glycaemic and insulinaemic homeostasis	Postprandial glucose; insulin/C-peptide responses; glycaemic index; fasting blood glucose; fasting insulin; HOMA-IR; HbA1c; GLP-1; GIP; fructosamine	Most studies comparing sourdough with yeast-fermented bread reported lower postprandial glycaemic and/or insulinaemic responses with sourdough. In contrast, results for the general effect of fermentation and for longer-term bread consumption were inconsistent, with most longer-term studies showing no significant improvement in fasting glycaemic markers.	Low for a beneficial effect of sourdough compared with yeast-fermented bread; very low for the general effect of fermentation; very low for longer-term bread consumption effects.	Current evidence suggests a possible beneficial effect of sourdough fermentation on postprandial glycaemic response, but the evidence is insufficient and inconsistent to substantiate a general health claim related to fermented bread consumption and glycaemic control in healthy adults.
**Review type**	**Search date range**	**Risk-of-bias tool**		**Comparator**		**Limitations**		
Systematic review	Sept 2023 – Jan 2025 (fermented cereals focus)	Cochrane RoB2 ("Revised Cochrane risk-of-bias tool for randomized trials")		Unfermented equivalent products where available (e.g., unfermented bread, pasta, porridge); PICO-defined		Small number of eligible studies with heterogeneous design and predominantly small sample sizes; strong geographical concentration in European (particularly Nordic) populations limits external validity; heterogeneous definition of "sourdough bread" across studies; longer-term/habitual-consumption effects remain unclear due to lack of dose-response and longer-duration trials		

**E5. Yilmaz *et al.* (2025). Fermented dairy product consumption and blood lipid levels in healthy adults: a systematic review (5).**								
**Number of papers**	**Meta-analysis**	**Type of FF most studied**	**Population**	**Main outcome**	**Specific outcomes**	**Main Results**	**Evidence**	**Comments / EFSA allegation**
14 controlled interventions (PICO), 37 non-controlled interventions (PIO), and 17 observational studies	No	Fermented dairy products: Yogurt, kefir, fermented milk, cheese	Healthy adults	Blood lipid profile and cardiometabolic effects	HDL cholesterol; LDL cholesterol; total cholesterol; triglycerides	A few studies reported modest reductions in total cholesterol and LDL cholesterol, or improved LDL/HDL ratios, particularly for yogurt and kefir, but interventional findings were largely inconsistent.	Evidence is inconsistent and overall insufficient.	A clear and consistent causal relationship cannot be concluded; overall, evidence is neither convincing nor sufficient because of heterogeneous designs, high risk of bias, inadequate product characterization, and limited mechanistic data.
**Review type**	**Search date range**	**Risk-of-bias tool**		**Comparator**		**Limitations**		
Systematic review	Inception – Dec 2024	Cochrane RoB2 (controlled trials only)		No/lower/less-frequent intake of fermented dairy		Comprehensive product characterisation is frequently lacking, and non-standardised dosing/microbial content limits robust dose–response conclusions		

E6. Mayo *et al.* (2025). Impact of yogurt consumption on bone health markers in adults with or without osteoporosis: a systematic review and meta-analysis (6).								
**Number of papers**	**Meta-analysis**	**Type of FF most studied**	**Population**	**Main outcome**	**Specific outcomes**	**Main Results**	**Evidence**	**Comments / EFSA allegation**
12 observational studies (cohort, case-control and cross-sectional studies)	Yes, 6 papers used for meta-analysis	Yogurt	Healthy adults and adults with osteoporosis	Bone health markers	Bone mineral density<br>Transition in bone status (e.g., from osteoporosis to osteopenia)<br>Incidence of pathological fractures (hip, spine, wrist)<br>Serum calcium and alkaline phosphatase levels<br>Bone turnover markers<br>Quality of life measurements<br>Body mass index	Some studies reported modest improvements in bone biomarkers and bone-related outcomes, but pooled evidence did not support a meaningful effect on fracture prevention or bone mineral density.	Low for the main quantitative outcomes.	The overall evidence remains inconclusive and neither convincing nor sufficient; yogurt does not currently support a cause-and-effect relationship for fracture prevention or meaningful improvement in bone mineral density.
**Review type**	**Search date range**	**Risk-of-bias tool**		**Comparator**		**Limitations**		
Systematic review + meta-analysis	1 Jan 1970 – 31 Oct 2023, update to 31 Dec 2023	Newcastle-Ottawa Scale		No yogurt consumption, or placebo		Yes — Section 4.1: observational-design confounding, small hip-fracture subset (3 studies), yogurt-characterisation variability		

E7. Harsa *et al.* (2025). The effects of Lactobacillus and/or Bifidobacterium in fermented foods on cognitive health: a systematic review (7).								
**Number of papers**	**Meta-analysis**	**Type of FF most studied**	**Population**	**Main outcome**	**Specific outcomes**	**Main Results**	**Evidence**	**Comments / EFSA allegation**
21 studies (8 interventional -13 observational studies)	No	Lactobacillus and/or Bifidobacterium in Fermented Foods: milk & skim milk fermented, soy based fermented. Mainly dairies in general (or specified: kefir, cheese..)	Healthy adults, including some adults with mild cognitive impairment	Cognitive health	Attention; memory; executive function; language; visuospatial abilities; hippocampal-dependent relational memory; working memory; declarative memory; delayed memory; verbal learning; general cognition; dementia risk	Some studies suggest promising cognitive benefits, but findings were heterogeneous across food types, study designs, and cognitive endpoints. A clear and consistent causal relationship could not be established.	Evidence is heterogeneous and limited.	A clear and consistent causal relationship between the consumption of a food fermented by L&B and a cognitive benefit cannot be concluded
**Review type**	**Search date range**	**Risk-of-bias tool**		**Comparator**		**Limitations**		
Systematic review	1 Jan 1970 – 31 Aug 2023	Muka et al./Cochrane methodology (RoB2-style domains)		Not directly quoted in eligibility criteria		Studies are limited by lack of appropriate controls, inconsistent reporting of demographic/health confounders, and absence of sex-specific analyses		

**S1. Künili *et al.* (2025). Bioactive compounds in fermented foods: a systematic narrative review (8).**								
**Number of papers**	**Meta-analysis**	**Type of FF most studied**	**Population**	**Main outcome**	**Specific outcomes**	**Main Results**	**Evidence**	**Comments / EFSA allegation**
50 (1 observational; 49 intervention)	No	Varied, mostly fermented dairy	18–70 y	Catalogue of fermentation derived bioactive compounds that linked to significant health outcomes	Cardiovascular/BP; lipid metabolism/obesity; glucose regulation; immune modulation/inflammation; neuroprotective/cognitive; liver function; other outcomes	31 bioactive compounds identified; studies grouped by health domain	Evidence strength not graded (no EFSA evidence statements); detailed food characterisation not included	Not suitable for EFSA allegation (purpose is compound catalogue; no EFSA grading)
**Review type**	**Search date range**	**Risk-of-bias tool**		**Comparator**		**Limitations**		
Systematic narrative review	Aug 2023 – Dec 2024	None (compound catalogue; no formal RoB)		PICO-structured comparator tables (Section 2.3.1.3)		Many studies lack mechanistic/biomarker data and strain-level characterisation, limiting causal inference and reproducibility		

S2. Keyvan *et al.* (2025). Vitamins formed by microorganisms in fermented foods: effects on human vitamin status—a systematic narrative review (9).								
**Number of papers**	**Meta-analysis**	**Type of FF most studied**	**Population**	**Main outcome**	**Specific outcomes**	**Main Results**	**Evidence**	**Comments / EFSA allegation**
18 (10 interventional, including 3 RCT-cross over, 6 observational	No	Natto; cheese; tea; fermented milk; coffee	Healthy adults Patients taking warfarin for prevention of thrombosis	Vitamin status (K, B3, B5, B6, B9, B12)	Bone health	The most consistent positive findings concerned vitamin K, particularly with natto, and to a lesser extent certain cheeses. Evidence for B vitamins was much more limited and less consistent.	Encouraging but limited; evidence is stronger for vitamin K than for B vitamins.	Fermented foods may contribute to vitamin intake through microbial production, but major gaps remain regarding bioavailability, vitamin forms, food-matrix effects, microbial strains, long-term outcomes, and safety.
**Review type**	**Search date range**	**Risk-of-bias tool**		**Comparator**		**Limitations**		
Systematic narrative review	1970–2024 initial; update Sept 2023–Dec 2024	Not reported		Generic comparator conditions		Evidence is concentrated in elderly/postmenopausal populations, limiting generalisability; individual gut-microbiota variation may affect vitamin K metabolism		

**S3. Nikolaou *et al.* (2026). Effects of sourdough-or regular-bread fermentation, and phytate reduction on iron bioavailability, absorption, and iron status in humans: a systematic review of intervention studies (10)**								
**Number of papers**	**Meta-analysis**	**Type of FF most studied**	**Population**	**Main outcome**	**Specific outcomes**	**Main Results**	**Evidence**	**Comments / EFSA allegation**
8 interventions	No	bread	Healthy or iron deficient	Iron bioavailability	Ferritin, total body iron, haemoglobin	Acute postprandial studies increased non-haem iron bioavailability (especially in low-phytate breads)/ long-term trials did not improve (or even decreased), ferritin and total body iron. Phytate reduction combined with iron fortification showed positive effects on haemoglobin or prevented iron depletion.	Results were inconclusive. Improvement only in deficient people (women, children) when fortified foods are used (with amaranth or iron)	Despite convincing mechanistic evidence that the sourdough-fermentation process in bread fabrication improves iron bioavailability, through reduction of phytate, no human studies address this research question with the appropriate control and study quality
**Review type**	**Search date range**	**Risk-of-bias tool**		**Comparator**		**Limitations**		
Systematic review of intervention studies	To 31 Aug 2023, expanded to 31 Dec 2024	Cochrane RoB2		High-phytate bread (study-specific)		High risk of bias across included studies (intervention deviations, missing outcome data, selective reporting) limits confidence in the synthesis		

S4. Santa *et al.* (2025). Health benefits of ethnic fermented foods (11).								
**Number of papers**	**Meta-analysis**	**Type of FF most studied**	**Population**	**Main outcome**	**Specific outcomes**	**Main Results**	**Evidence**	**Comments / EFSA allegation**
125 (67 interventional, 58 observational	No	Ethnic fermented foods, including amazake, boza, buttermilk, injera, kimchi, miso, natto, and tempeh	All age groups and health states; studies were concentrated mainly in Asia and Africa	Gut health, immune modulation, metabolic benefit, cognitive function, cardiovascular, bone, oral, eye, skin health and antimicrobial effect	Specific to each outcome, not the same for one syndrome	This systematic narrative review highlights the consistent health benefits of EFF reported in 125 human studies across diverse regions over five decades	These benefits arise not only from raw ingredients or probiotic content, but from the fermentation process itself, which enhances nutrient bioavailability, reduces antinutrients, and generates bioactive compounds such as amino acids, peptides, and vitamins (K2, B12, riboflavin, folate).	The contributions from the fermentation process itself may be the most critical, as it can release or transform the bioactive compounds that are otherwise inaccessible in the raw material or absent from starter cultures alone. This highlights the unique role of fermentation in generating health-relevant components that would not emerge without this biochemical transformation.<br>his strategy consequently strongly biased the review towards the beneficial side of the functionality of EFF.
**Review type**	**Search date range**	**Risk-of-bias tool**		**Comparator**		**Limitations**		
Comprehensive narrative review	1970–2025	None (N/A)		Not formally defined		Data extraction captured only beneficial outcomes (neutral/detrimental findings excluded), which the authors note strongly biases the review towards positive findings		

**S5A. Paveljšek *et al.* (2025), A systematic review of prospective evidence linking non-alcoholic fermented food consumption with lower mortality risk (12).**								
**Number of papers**	**Meta-analysis**	**Type of FF most studied**	**Population**	**Main outcome**	**Specific outcomes**	**Main Results**	**Evidence**	**Comments / EFSA allegation**
52 studies – Prospective cohort studies	No	Total FF and specific FFs (dairy products, milk products, yogurt, cheese, fermented soy, miso, Natto, fermented vegetables, fermented meat, bread, chocolate and cocoa	Prospective cohort populations	Mortality risk	All-cause mortality; cardiovascular mortality; cancer mortality; gastrointestinal cancer mortality; lung cancer mortality; reproductive cancer mortality	Habitual consumption of certain fermented foods was associated with modest reductions in all-cause and cause-specific mortality, with the most consistent signals observed for some fermented dairy products, fermented milk, yogurt, natto, and chocolate/cocoa. Findings varied substantially by food category and mortality outcome	Evidence ranged from very low to high depending on the fermented food category and mortality outcome.	Habitual consumption of certain fermented foods may be associated with modest reductions in mortality risk, but the current evidence remains insufficient to support EFSA approved health claims.
**Review type**	**Search date range**	**Risk-of-bias tool**		**Comparator**		**Limitations**		
Systematic review (prospective cohort)	1970–2025 (search 2023, updated 15 Jan 2025)	Newcastle-Ottawa Scale		N/A (observational, no intervention comparator)		Several FF categories are sparsely represented with non-standardised exposure measures; publication-bias testing was not feasible for most categories		

S5B. Matalas *et al.* (2025). Fermented foods consumption, all-cause, and cause-specific mortality: a meta-analysis of prospective cohort studies (13).								
**Number of papers**	**Meta-analysis**	**Type of FF most studied**	**Population**	**Main outcome**	**Specific outcomes**	**Main Results**	**Evidence**	**Comments / EFSA allegation**
50 prospective cohort studies	Yes	Yogurt; fermented milk; cheese; unspecified fermented dairy products; chocolate products; miso products	Prospective cohort populations	Mortality risk	All-cause mortality; cardiovascular mortality; cancer mortality; gastrointestinal cancer mortality; lung cancer mortality; reproductive cancer mortality	Meta-analytic results suggest that fermented products are generally protective or neutral in relation to mortality risk, with modest pooled associations for some fermented dairy products and category-specific outcomes. Heterogeneity remained considerable for several food categories and endpoints.	Effect sizes were generally modest; interpretation is limited by heterogeneity across foods and outcomes.	Existing evidence is insufficient to define the long-term health implications of fermented food consumption, although mortality and cause-specific mortality remain relevant global markers of dietary impact on health.
**Review type**	**Search date range**	**Risk-of-bias tool**		**Comparator**		**Limitations**		
Meta-analysis of prospective cohort studies	Since 2023, updated to 31 Mar 2025	Newcastle-Ottawa Scale		N/A (observational)		Yes — Section 5: dietary-assessment-instrument limitations, food-category heterogeneity		

**S6. Humblot *et al.* (2025). A scoping review of the health effects of fermented foods in specific human populations and their potential role in precision nutrition: current knowledge and gaps (14).**								
**Number of papers**	**Meta-analysis**	**Type of FF most studied**	**Population**	**Main outcome**	**Specific outcomes**	**Main Results**	**Evidence**	**Comments / EFSA allegation**
47 (27 interventional, 37 observational)	22 papers	Fermented milk (yogurt, cheese, butter)<br>Bread<br>Soy (Natto, Miso)<br>Apple cider vinegar<br>chocolate<br>coffee	Adults Healthy/pregnant/overweighed/T2D/MetS/mildly hypercholesterolemic/ cancer	MetS, Psychological disorders, Cancer, Gut barrier leaking, Muscle loss	Metabolic syndrome-related outcomes; psychological outcomes; cancer-related outcomes; gut barrier function; muscle loss	Fermented foods may have potential in tailored nutritional strategies, but the literature is heterogeneous and often based on a limited number of studies per subgroup or outcome. Reported differences between populations suggest response heterogeneity, but the determinants of this variability remain insufficiently resolved.	Evidence is suggestive but fragmented, with many outcome-population combinations supported by only a few studies.	The analysis of the existing literature in the field of the differential response of populations shows that a heterogeneity of response indeed exists. However, factors of variability within populations can be intertwined; differences observed between stratified populations can be relatively small, with the size of the population subgroups even smaller due to stratification;<br>evaluation of the consumption (type, frequency and amount) of FF is generally poorly described).

**Review type**	**Search date range**	**Risk-of-bias tool**	**Comparator**	**Limitations**
Scoping review	1970–Aug 2023, final update to 31 Mar 2025	None (scoping review)	Absence/lower consumption of FF, or non-FF equivalent	Confounding factors and inconsistent exposure definitions (e.g., “a cup of coffee”) limit comparability of stratified analyses

S7. Assunção *et al.* (2026). Are fermented foods safe for human health? A systematic analysis of the literature (15)								
**Number of papers**	**Meta-analysis**	**Type of FF most studied**	**Population**	**Main outcome**	**Specific outcomes**	**Main Results**	**Evidence**	**Comments / EFSA allegation**
123 studies (per published PRISMA flow diagram)	No	Broad range across major fermented food categories (dairy, meat and fish, fruits and vegetables, beverages, legumes, cereals, etc.); dairy and fermented meat products most frequently implicated in reported outbreaks	General population, including vulnerable groups (elderly, children, pregnant/breastfeeding women); no age, gender, or health-status restriction	Occurrence of microbiological and chemical hazards in fermented foods, associated exposure/risk, and reported foodborne outbreaks	Microbiological hazards (e.g., Salmonella, pathogenic E. coli, Listeria monocytogenes, Campylobacter jejuni, Clostridium botulinum); chemical hazards (mycotoxins, heavy metals, polycyclic aromatic hydrocarbons, acrylamide); reported foodborne outbreaks	Identified hazards were predominantly linked to contaminated raw materials, inadequate processing, or post-process/environmental contamination rather than fermentation itself; most risk metrics indicated low concern under typical consumption, though specific cheeses and cereal-based foods showed higher chemical exposure	Not Applicable — no formal quality appraisal or EFSA benefit-certainty framework applied; heterogeneous study designs (outbreak investigations, case reports, observational studies, exposure assessments) precluded use of a single standardised tool	Not Applicable (safety review; not framed as a health-claim substantiation). Overall conclusion: fermented foods are generally safe for human consumption when produced under appropriate hygienic and technological conditions
**Review type**	**Search date range**	**Risk-of-bias tool**		**Comparator**		**Limitations**		
Systematic scoping review (safety focus)	1 Jan 1970 – 31 Dec 2024	Not Applicable — no formal quality appraisal applied; heterogeneous study designs (outbreak investigations, case reports, observational studies, exposure assessments) precluded use of a single standardised RoB tool		Not Applicable — comparator dimension (absence of fermented food consumption) explicitly not considered; PECO framework used for eligibility, but review is hazard/incident-occurrence-based rather than comparator-based		Heterogeneous study designs (outbreak investigations, case reports, observational studies, quantitative exposure assessments) precluded a single standardised quality-appraisal tool, so not all included studies contribute equally to the conclusions; publication/reporting bias in outbreak detection and surveillance varies by country; quantitative risk estimates (particularly for chemical hazards) draw on studies using different populations, dietary surveys, analytical methods, and exposure models, limiting direct cross-study comparison; evidence base weighted toward established food categories and may underrepresent traditional, household, or emerging fermented foods		

S8. Berkel Kasikci *et al.* (2025). A systematic review on the health effects of fermented wheat germ extract with emphasis on cancer (16).								
**Number of papers**	**Meta-analysis**	**Type of FF most studied**	**Population**	**Main outcome**	**Specific outcomes**	**Main Results**	**Evidence**	**Comments / EFSA allegation**
6 interventional	No	Fermented wheat germ extract	Adults with cancer or inflammatory conditions	Anticancer and anti-inflammatory effects	Survival; quality of life; cancer-related outcomes	Potential bioactive constituents of FWGE, particularly benzoquinones, peptides, and phenolic compounds, as mediators of its anticancer and anti-inflammatory properties. Among its proposed mechanisms, FWGE may suppress cancer cell proliferation by disrupting the glucose-related metabolic pathways.	While the findings suggest that FWGE may possess therapeutic potential, especially in oncology, the strength of evidence remains limited.	Current evidence is insufficient for firm conclusions, and well-designed clinical trials are needed
**Review type**	**Search date range**	**Risk-of-bias tool**		**Comparator**		**Limitations**		
Systematic review	Not stated	Newcastle-Ottawa Scale (observational) + Cochrane RoB2 (interventional)		Not directly quoted		Small sample sizes, short study durations, and methodological weaknesses limit generalisability; no universal production standard exists for FWGE		

S9. Sar *et al.* (2025). A systematic review of health promoting effects of consumption of whey-based fermented products on adults (17).								
**Number of papers**	**Meta-analysis**	**Type of FF most studied**	**Population**	**Main outcome**	**Specific outcomes**	**Main Results**	**Evidence**	**Comments / EFSA allegation**
12 interventional	No	Whey-based fermented products	Adults across diverse groups, including healthy individuals, hypercholesterolemic participants, metabolic syndrome, haemodialysis patients, men with urinary tract symptoms, and H. pylori-infected volunteers	Health effects of fermented whey products	Urinary tract health; immune function; muscle mass; glycaemia; lipid profile; oxidative stress; inflammation; gastrointestinal health; nutritional status	Fermented whey products showed promising effects across multiple physiological systems, including metabolic regulation, gastrointestinal and cardiovascular health, immune function, and oxidative stress. Most interventions were well tolerated, but findings were heterogeneous across products and study populations.	Promising but limited by small samples, short durations, and heterogeneity of interventions and outcomes.	Clinical studies indicate positive effects on metabolic regulation, gastrointestinal and cardiovascular health, and immune function. However, the overall quality and consistency of the evidence remain limited. Small sample sizes, brief intervention periods, and lack standardization in both the composition and dosage of the interventions. Methodological issues such as absence of randomization, insufficient blinding, and selective outcome reporting further weaken the reliability of findings. Mechanistic investigations are also needed to better understand the biological pathways involved and to assess potential variability in individual responses
**Review type**	**Search date range**	**Risk-of-bias tool**		**Comparator**		**Limitations**		
Systematic review	1 Jan 1970 – 31 Dec 2024	Risk of Bias 2 (RoB2)		Placebo or non-fermented dairy		Small sample sizes, brief intervention periods, lack of standardisation in composition/dosage		

Supplementary Table S2. Tabular representation of Strategic Roadmap. The table beneath informs the Strategic Roadmap as shown in Figure 1 in the main text. The table links certain EFSA dossier Domains (created for ease of categorisation but loosely based on EFSA requirements) with Knowledge gaps and Roadmap objectives. ST: short-term; MT: medium-term; LT: long-term; R#: research roadmap objective number; P#: policy roadmap objective number.

Code	Domain	Subdomain	Framework Header	Focus	Description	Linked Knowledge Gap[#]	Roadmap Objectives*
**F1A**	Food Characterisation	Raw Materials & Composition	Baseline Food Matrix Profile	Matrix, nutrients, raw materials	Clear classification of starting raw materials (e.g., dairy, cereal, legume) and baseline nutritional profiles before fermentation.	Incomplete characterisation of raw materials (HIGH).	ST-R1, ST-P2.
**F2A**	Food Characterisation	Food Diversity & Cultural Scope	Inclusion of Traditional FFs	Non-dairy, artisanal, regional	Expanding research beyond dairy to traditional, non-dairy, and artisanal fermented foods.	Lack of diversity in FFs studied (HIGH).	MT-R7, MT-P10.
**F3A**	Food Characterisation	Manufacturing Process	Fermentation Parameters	Time, temp, pH, oxygen	Standardised reporting of critical process parameters controlling the fermentation.	Inadequate reporting of fermentation parameters (HIGH).	ST-R1, ST-P3.
**F3B**	Food Characterisation	Manufacturing Process	Substrate-Microbe Combinations	Starter cultures, matrix-microbe	Understanding how specific microbes interact with substrates to generate targeted bioactives.	Limited understanding of substrate-microbe interactions (MED-HIGH).	MT-R8, LT-R13.
**F3C**	Food Characterisation	Manufacturing Process	Reporting Transparency	CONSORT-style, minimum standards	Harmonised frameworks and open reporting of proprietary fermentation processes.	No harmonised fermentation reporting standards (HIGH); Proprietary constraints limit transparency (LOW-MED).	ST-R1, MT-P8.
**F4A**	Food Characterisation	End-Product Profiling	Microbial Identity & Metabolites	Strain-level, viability, bioactives	Comprehensive profiling of fermentation-derived metabolites and strain-level microbial identity.	Lack of comprehensive end-product composition profiling (HIGH).	ST-R1, ST-P1, ST-P2.
**F4B**	Food Characterisation	End-Product Profiling	Intracategory Variability	Batch consistency, regional differences	Explicit documentation of variability across different batches and regional manufacturing differences.	Poor documentation of intracategory variability (MEDIUM).	ST-R3, ST-P3.

**F5A**	Food Characterisation	Stability & Delivered Exposure	Shelf-life Stability	Viability retention, metabolite stability	Tracking microbial viability and metabolite retention from the point of production through to shelf life.	Lack of microbe viability/metabolite stability data over shelf life (HIGH).	ST-R1, ST-P2.
**F5B**	Food Characterisation	Stability & Delivered Exposure	Consumed Dose Measurement	Point-of-intake dose	Aligning initial product characterisation with the exact dose delivered at the moment of consumption.	Poor alignment between product characterisation and consumed dose (HIGH).	ST-R4, ST-P2.
**A1A**	Analytical Methods	Analytical Methodologies	Validated Analytical Tools	Standardised methods, quantification	Development and utilization of harmonised, validated methods for quantifying specific microbes and metabolites.	Lack of harmonised, validated analytical frameworks (HIGH).	ST-R3, MT-P8.
**A1B**	Analytical Methods	Analytical Methodologies	Hypothesis-Aligned Measures	Pre-specified bioactives	Ensuring analytical measures and biomarker selection directly align with the mechanistic hypotheses of the study.	Analytical measures not aligned with mechanistic hypotheses (MEDIUM).	ST-R3, ST-R4.
**D1A**	Dietary Assessment	Exposure Tracking	Precision in Dietary Recall	Validated FFFQ, AI assessment	Upgrading tracking instruments like the Fermented Food Frequency Questionnaire (FFFQ) to reduce recall misclassification.	High misclassification from FFQ/recall (HIGH).	ST-R2, ST-P4.
**D1B**	Dietary Assessment	Exposure Tracking	Subcategory Differentiation	Explicit classification, specific intake	Classifying FFs beyond broad labels like "fermented dairy" to accurately capture biological exposure differences.	Poor differentiation of FF subcategories in surveys (HIGH).	ST-R2, ST-P4.
**D1C**	Dietary Assessment	Exposure Tracking	Consumer Identification	Labelling, public awareness	Enhancing consumer recognition of specific FFs through standardized labelling indicating live vs. non-viable cultures.	Participants may not recognise fermented foods (MEDIUM).	ST-P3, Cross-cutting Policy.
**H1A**	Human Evidence	Specificity & Magnitude of Effect	Specificity of Clinical Outcomes	Attributable effects, unique outcomes	Establishing effects specifically attributable to the fermentation process rather than overlapping raw matrix effects.	Poor specificity of effect across FFs (HIGH).	ST-R4, MT-R8.

**H1B**	Human Evidence	Specificity & Magnitude of Effect	Clinical Relevance	Effect sizes, magnitude	Designing well-powered trials to determine if physiological changes consistently cross clinical relevance thresholds.	Effect sizes are small/modest (MEDIUM).	LT-R9.
**H2A**	Human Evidence	Dose-Response	Minimum Effective Dose	Dose-response, optimal intake	Evaluating different intake amounts to establish clear dose-response relationships or minimum effective microbial loads.	Lack of dose-response evidence (HIGH).	LT-R9, LT-P11.
**H3A**	Human Evidence	Study Design & Controls	Comparator Selection	Matched controls, pasteurised	Integrating appropriate non-fermented or pasteurised comparators to properly isolate active fermentation effects.	Poor comparators (HIGH).	ST-R4, MT-P8.
**H3B**	Human Evidence	Study Design & Controls	Methodological Consistency	Harmonised trials, study design	Standardising clinical trial designs globally to reduce heterogeneity and enable structured meta-analyses.	Extremely high heterogeneity across studies (HIGH).	MT-P8, MT-P9.
**H4A**	Human Evidence	Population & Sustainability	Long-term Outcome Tracking	Follow-up, chronic diseases	Tracking the sustained effects of interventions on chronic disease endpoints over extended periods.	Lack of longterm data (MEDIUM).	LT-R9.
**H4B**	Human Evidence	Population & Sustainability	Diverse Demographics	Vulnerable groups, global cohorts	Including children, the elderly, and nutritionally deficient subgroups to ensure broad population applicability.	Underrepresentation of vulnerable populations (MEDIUM).	MT-R7, MT-P10.
**M1A**	Mechanisms	Bioavailability	In Vivo Systemic Exposure	Human absorption, pharmacokinetics	Providing direct human pharmacokinetic evidence of absorption and bioavailability for fermentation-derived compounds.	Lack of human evidence for enhanced bioavailability (HIGH).	MT-R5, MT-P6.
**M1B**	Mechanisms	Bioavailability	Integrated Determinants	Matrix effects, microbiome integration	Designing trials that jointly evaluate how the food matrix, host microbiome, and	Determinants of bioavailability not studied together (MEDIUM).	MT-R6.

					fermentation conditions impact bioavailability.		
**M2A**	Mechanisms	Causal Linkages	Biomarker & Pathway Validation	Mechanistic tracking, human biomarkers	Integrating mechanistic biomarkers into human trials to explicitly validate hypothesized functional pathways.	Mechanistic human studies lack biomarkers or pathway validation (HIGH).	MT-R5, LT-R11.
**M2B**	Mechanisms	Causal Linkages	Direct Physiological Linkages	Causal chains, bioactive levels	Linking measured changes in specific fermentation bioactives directly to human physiological outcomes.	Lack of linkages between bioactive levels and physiological effects (HIGH).	MT-R8.
**M3A**	Mechanisms	Vitamins	Human Vitamin Status Contribution	B12, K2, vitamer production	Demonstrating that the consumption of FF-derived vitamins directly yields improved human nutritional status.	Limited human studies linking FF-derived vitamins to status (HIGH).	MT-R5, LT-P12.
**M3B**	Mechanisms	Vitamins	Optimisation of Vitamer Production	Strain capabilities, vitamin synthesis	Characterising the precise strains and conditions that safely optimise vitamin yield during food fermentation.	Poorly characterised mechanisms of vitamin production (MEDIUM).	LT-P13.
**S1A**	Safety	Clinical Tolerability	Systematic Adverse Event Tracking	Safety endpoints, adverse events	Moving past historical "GRAS" assumptions by actively monitoring and reporting adverse events during clinical trials.	Inconsistent safety reporting in trials (HIGH).	ST-P5.
**S2A**	Safety	Vulnerability & Risk	Safety Profiles in Vulnerable Groups	Sensitive populations, tolerability	Generating explicit safety and tolerability data for immunologically or nutritionally vulnerable demographic groups.	Limited safety data in vulnerable groups (MEDIUM).	MT-R7, MT-P10.
**S2B**	Safety	Vulnerability & Risk	Contamination & Hazard Surveillance	Real-world hazards, biogenic amines	Integrating the monitoring of foodborne pathogens and undesirable compounds (like biogenic amines) into safety endpoints.	Clinical studies overlook realworld contamination risks (LOW-MEDIUM).	ST-P5.

**Y1A**	Synthesis & Integration	Regulatory Submissions	Totality of Evidence Grading	Health claims, causality	Compiling unified evidence packages resolving the current gap where evidence is deemed "neither convincing nor sufficient".	Mechanistic evidence is insufficient for regulatory requirements (HIGH).	MT-P6, LT-P12.
**Y1B**	Synthesis & Integration	Regulatory Submissions	Global FF Databases	Open-access repositories, data sharing	Establishing open-access datasets combining strain, metabolite, and nutritional data universally.	No fermented-food composition databases (MEDIUM-LOW).	LT-R10, MT-P7, MT-P9.
**Y2A**	Synthesis & Integration	Precision Nutrition	Tailored Dietary Integration	Predictive models, targeted advice	Utilising microbiome, metabolomic, and genetic data to create targeted recommendations and precision functional FFs.	*(Long-term translational goal)* Integration into preventive healthcare strategies and dietary guidelines.	LT-R12, LT-P11, LT-P13, LT-P14.

#Priority levels as derived from Knowledge gaps are presented in parentheses.

*Research Roadmap objectives: 1. Harmonisation of fermented food characterisation, 2. Improvement of exposure assessment tools, 3. Strengthening analytical and methodological rigor, 4. Refinement of study design and comparators, 5. Mechanistic validation in humans, 6. Integration of microbiome and inter-individual variability, 7. Expansion of research scope and populations, 8. Linking composition → mechanism → outcome, 9. Large-scale, long-term clinical trials, 10. Development of global fermented food databases, 11. Biomarker and exposure standardisation, 12. Precision nutrition and personalised responses, 13. Exploration of novel mechanisms and bioactives; Policy Roadmap objectives: 1. Establish harmonised definitions and classification systems, 2. Develop regulatory guidance for fermented foods characterisation, 3. Standardise labelling and transparency requirements, 4. Integrate fermented foods into dietary surveillance systems, 5. Strengthen safety monitoring frameworks, 6. Develop regulatory frameworks for bioactive compounds and microbes, 7. Create global fermented food composition databases, 8. Standardise clinical trial frameworks for fermented foods, 9. Enable data sharing and research infrastructure, 10. Address population diversity and equity, 11. Establish evidence-based dietary recommendations for fermented foods, 12. Enable health claim approvals and regulatory recognition, 13. Support development of targeted and precision fermented foods, 14. Integrate fermented foods into preventive healthcare strategies.

References

1. Mukherjee A, Farsi DN, Garcia-Gutierrez E, Akan E, Millan JAS, Angelovski L, et al. Impact of fermented foods consumption on gastrointestinal wellbeing in healthy adults: a systematic review and meta-analysis. Front Nutr. 2025;12:1668889. doi: 10.3389/fnut.2025.1668889.
2. Hyseni B, Papadimitriou K, Issa A, Tonay AN, Ergün BG, Gonzalez-Domenech CM, et al. The role of fermented foods in managing food allergies in children and adults: a systematic review. Frontiers in Nutrition. 2026;Volume 12 - 2025. doi: 10.3389/fnut.2025.1689636.
3. Akpınar A, Markiewicz LH, Harsa H, Paveljšek D, Domínguez-Soberanes J, Agirbasli Z, et al. Efficacy of fermented foods for the prevention and treatment of bacterial vaginosis and vulvovaginal candidiasis. Front Nutr. 2025;12:1658988. doi: 10.3389/fnut.2025.1658988.
4. Starowicz M, Domínguez-Soberanes J, Garcia-Gutierrez E, Arslan-Tontul S, Burton-Pimentel KJ, Vidović B, et al. Effects of sourdough and yeast fermented breads consumption on glycaemia in healthy populations: a systematic review. Frontiers in Nutrition. 2026;Volume 13 - 2026. doi: 10.3389/fnut.2026.1910648.
5. Yilmaz B, Alvanoudi P, Kalogeropoulou A, Santa D, Bulmuş-Tüccar T, Nikolaou A, et al. Fermented dairy product consumption and blood lipid levels in healthy adults: a systematic review. Front Nutr. 2025;12:1651134. doi: 10.3389/fnut.2025.1651134.
6. Mayo B, Malagón-Rojas J, Agahi F, Frias J, Künili İ E, Kütt ML, et al. Impact of yogurt consumption on bone health markers in adults with or without osteoporosis: a systematic review and meta-analysis. Front Nutr. 2025;12:1660505. doi: 10.3389/fnut.2025.1660505.
7. Harsa HS, González Domenech CM, Prvulović M, Agirbasli Z, Bagherzadehsurbagh E, Simeunović V, et al. The effects of Lactobacillus and/or Bifidobacterium in fermented foods on cognitive health: a systematic review. Front Nutr. 2025;12:1682419. doi: 10.3389/fnut.2025.1682419.
8. Künili IE, Akdeniz V, Akpınar A, Budak Ş, Curiel JA, Guzel M, et al. Bioactive compounds in fermented foods: a systematic narrative review. Front Nutr. 2025;12:1625816. doi: 10.3389/fnut.2025.1625816.
9. Keyvan E, Adesemoye E, Champomier-Vergès MC, Chanséaume-Bussiere E, Mardon J, Nikolovska Nedelkoska D, et al. Vitamins formed by microorganisms in fermented foods: effects on human vitamin status-a systematic narrative review. Front Nutr. 2025;12:1653666. doi: 10.3389/fnut.2025.1653666.
10. Nikolaou A, Assunção R, Cvetkovic B, Fardet A, Gamero A, GANDÍA M, Mojsova S, Yilmaz B, Kütt M-L, Santa D. Effects of sourdough-or regular-bread fermentation, and phytate reduction on iron bioavailability, absorption, and iron status in humans: a systematic review of intervention studies. Frontiers in Nutrition. 2026;13:1778997.
11. Santa D, Huch M, Stoll DA, Cunedioglu H, Priidik R, Karakaş-Budak B, et al. Health benefits of ethnic fermented foods. Front Nutr. 2025;12:1677478. doi: 10.3389/fnut.2025.1677478.
12. Paveljšek D, Pertziger E, Fardet A, Panagiotakos DB, Savary-Auzeloux I, Adamberg S, et al. A systematic review of prospective evidence linking non-alcoholic fermented food consumption with lower mortality risk. Frontiers in Nutrition. 2025;Volume 12 - 2025. doi: 10.3389/fnut.2025.1657100.
13. Matalas A, Panagiotakos D, Fardet A, Savary-Auzeloux I, Chassard C, Praćer S, Vergères G, Paveljšek D. Fermented foods consumption, all-cause, and cause-specific mortality: a meta-analysis of prospective cohort studies. Frontiers in Nutrition. 2026;Volume 13 - 2026. doi: 10.3389/fnut.2026.1714437.
14. Humblot C, Alvanoudi P, Alves E, Assunçao R, Belovic M, Bulmus-Tuccar T, et al. A scoping review of the health effects of fermented foods in specific human populations and their potential role in precision nutrition: current knowledge and gaps. Frontiers in Nutrition. 2025;Volume 12 - 2025. doi: 10.3389/fnut.2025.1650633.

15. Assunção R, Angelovski L, Ciesarová Z, Russo P, Salvetti E, Sarand I, et al. Are Fermented Foods Safe for Human Health? A Systematic Analysis of the Literature. Frontiers in Nutrition, 2026. doi: 10.3389/fnut.2026.1905861
16. Berkel Kasikci M, Issa A, Baglam N, Dincer E, Gérard A, Kabakci S, et al. A systematic review on the health effects of fermented wheat germ extract with emphasis on cancer. Frontiers in Nutrition. 2025;Volume 12 - 2025. doi: 10.3389/fnut.2025.1677464.
17. Sar T, Bogovic Matijasic B, Danilovic B, Gamero A, Gandía M, Krausova G, et al. A systematic review of health promoting effects of consumption of whey-based fermented products on adults. Front Nutr. 2025;12:1651365. doi: 10.3389/fnut.2025.1651365.
